# Digital Twins for Clinical and Operational Decision-Making: Scoping Review

**DOI:** 10.2196/55015

**Published:** 2025-01-08

**Authors:** Vahid Riahi, Ibrahima Diouf, Sankalp Khanna, Justin Boyle, Hamed Hassanzadeh

**Affiliations:** 1 Australian e-Health Research Centre Commonwealth Scientific and Industrial Research Organisation Melbourne Australia; 2 Australian e-Health Research Centre Commonwealth Scientific and Industrial Research Organisation Brisbane Australia

**Keywords:** digital twin, health care, clinical decision-making, CDM, operational decision-making, ODM, scoping review

## Abstract

**Background:**

The health care industry must align with new digital technologies to respond to existing and new challenges. Digital twins (DTs) are an emerging technology for digital transformation and applied intelligence that is rapidly attracting attention. DTs are virtual representations of products, systems, or processes that interact bidirectionally in real time with their actual counterparts. Although DTs have diverse applications from personalized care to treatment optimization, misconceptions persist regarding their definition and the extent of their implementation within health systems.

**Objective:**

This study aimed to review DT applications in health care, particularly for clinical decision-making (CDM) and operational decision-making (ODM). It provides a definition and framework for DTs by exploring their unique elements and characteristics. Then, it assesses the current advances and extent of DT applications to support CDM and ODM using the defined DT characteristics.

**Methods:**

We conducted a scoping review following the PRISMA-ScR (Preferred Reporting Items for Systematic Reviews and Meta-Analyses extension for Scoping Reviews) protocol. We searched multiple databases, including PubMed, MEDLINE, and Scopus, for original research articles describing DT technologies applied to CDM and ODM in health systems. Papers proposing only ideas or frameworks or describing DT capabilities without experimental data were excluded. We collated several available types of information, for example, DT characteristics, the environment that DTs were tested within, and the main underlying method, and used descriptive statistics to analyze the synthesized data.

**Results:**

Out of 5537 relevant papers, 1.55% (86/5537) met the predefined inclusion criteria, all published after 2017. The majority focused on CDM (75/86, 87%). Mathematical modeling (24/86, 28%) and simulation techniques (17/86, 20%) were the most frequently used methods. Using *International Classification of Diseases, 10th Revision* coding, we identified 3 key areas of DT applications as follows: factors influencing diseases of the circulatory system (14/86, 16%); health status and contact with health services (12/86, 14%); and endocrine, nutritional, and metabolic diseases (10/86, 12%). Only 16 (19%) of 86 studies tested the developed system in a real environment, while the remainder were evaluated in simulated settings. Assessing the studies against defined DT characteristics reveals that the developed systems have yet to materialize the full capabilities of DTs.

**Conclusions:**

This study provides a comprehensive review of DT applications in health care, focusing on CDM and ODM. A key contribution is the development of a framework that defines important elements and characteristics of DTs in the context of related literature. The DT applications studied in this paper reveal encouraging results that allow us to envision that, in the near future, they will play an important role not only in the diagnosis and prevention of diseases but also in other areas, such as efficient clinical trial design, as well as personalized and optimized treatments.

## Introduction

### Background

Digital twins (DTs) are an emerging concept that has recently attracted the attention of researchers and engineers. The development of new technologies, such as virtual reality, blockchain, and the Internet of Things (IoT), is considered a key factor for progress in the DT research field [1]. However, the role of the recent boost in digital transformation and exponential growth in investments by giant tech companies cannot be neglected [2]. DTs have their roots in the field of engineering [3-6] but are quickly expanding to a wide range of applications, such as city planning [7-9], energy [10,11], retail [12,13], and health care [14,15]. Although DTs are an emerging concept, they integrate well-established technologies as underlying components. The origin of the DT goes back to the 1970s when NASA (National Aeronautics and Space Administration) used the “twin” concept by creating simulated environments (ie, mirrored systems) to monitor the spacecraft in the Apollo 13 program [16,17]. Although this instance may not be considered as an advanced DT today due to the lack of data exchange to allow continuous or periodic “twinning” of the digital to the physical [16,18], it represents a very good example of the potential of DT and what DTs can empower in various industries.

A DT of the city of Zurich, Switzerland [7], is a good example of a DT application. In this project, spatial 3D data is transferred to a DT to support urban planning decision-making, for example, simulating climate change and noise issues within urban plans [7]. Another example of a DT was built by Unilever PLC to increase the flexibility and efficiency of their production process [19]. They use DT systems to predict optimal process parameters for new formulations, execute simulations to identify the best operational conditions, and investigate complex what-if scenarios. DT systems have also been applied in the field of water management in Porto, Portugal [20], by creating a virtual representation of the city water network. This DT was developed to predict water quality and flooding issues, simulate and analyze burst pipe scenarios, and ensure the resilience of water infrastructure. The initial results revealed that the developed DT reduced water-supply failures by about 30% and decreased the time needed to repair burst pipes by 8% [20].

There have been several efforts to develop DTs for various purposes in health care. Clinical decision-making (CDM) and operational decision-making (ODM) are 2 of the most important decision-making processes in health care settings. In this context, CDM refers to the decisions made by health professionals in regard to direct health care delivery (diagnosis, test, intervention, etc) based on a patient’s health status and related clinical conditions, while ODM refers to the decisions made by managers and administrators after monitoring processes related to operations, such as patient waiting times and hospital revenue [21]. In subsequent sections of this paper, we present a scoping literature review to provide an up-to-date overview of the existing literature relevant to the application of DT systems in health care and to gauge the extent of the designed DT models for CDM and ODM purposes.

There have been several literature reviews on the applications of DTs in health care from different perspectives. Ahmadi-Assalemi et al [22] reviewed recent works in the field of precision health care and discussed the key enabling technologies (eg, IoT and cloud) of DTs. Hassani et al [2] provided a literature review that discussed the value of DTs in health care and proposed some key characteristics, including dynamic, real-time, and bidirectional data connections. Armeni et al [18] identified the opportunities as well as challenges regarding DT implementations in health care (eg, security and privacy, accessibility of the technology, and data collection and management). Elkefi and Asan [23] conducted a systematic review of studies that discussed the contribution of DTs to improving user experience in health care (eg, safety management or information management). They used *digital twin* and *health* as keywords and 17 papers were included in their review. Sun et al [1] provided a systematic review to explore the progress of prominent research on DT technology in medicine. They used the following terms: *digital twin*, *medicine*, *digital health*, and *virtual healthcare*. This resulted in the inclusion of 22 papers in their study after the screening process. They showed that the application of DTs to the cardiovascular system is an attractive area. Sheng et al [24] used structural topic modelling to analyze trends in *DT+healthcare* and their findings show that technology integration and practical applications, for example, IoT and artificial intelligence, are the main focus areas. Katsoulakis et al [25] conducted a scoping review on DTs for health. Using *digital twin* and *health* as search keywords, the authors selected 85 papers in their analysis. They first categorized the selected papers into 8 distinct categories based on their purpose and content, for example, DTs for biomarker and drug discovery as well as DTs in biomanufacturing. Then, they discussed the challenges hindering developments in this field, such as data privacy and security, computing infrastructure, and data quality and accuracy.

### This Paper

In this paper, we present a distinctive approach that distinguishes our work from existing literature reviews. The key distinctions are discussed herein. First, the health care industry and academia use various definitions, necessitating research consolidation to establish a unified understanding and ensure future research builds on robust foundations. This study conducts a systematic literature review and thematic analysis of 86 publications on DTs and offers a comprehensive analysis of elements and characterization of the concept, which is absent in existing review papers. This explicit definition of DTs, along with the identification of their elements and characteristics, enables researchers to understand what distinguishes a DT as a unique system, separate from other known ones. Second, DTs represent a burgeoning research field, particularly in health care, prone to misconceptions and misinterpretations. A significant gap in the literature lies in distinguishing established models, such as machine learning and 2D or 3D modeling from true DTs. This paper pioneers an effort to clarify that not all papers claiming to develop DTs genuinely do so. Our analysis reveals that some are simply predictive models or simulations, branded under the DTs trend. This trend risks undermining the integrity of the research. Thus, this paper aims to educate readers on discerning authentic DTs, emphasizing the essential components required for a system to qualify as such.

### What Is a DT?

#### Overview

There are similarities between DTs and other related technologies, such as simulation, predictive modeling, and 3D modeling, which leads to apparent misconceptions about their definition [2,26]. A summary of DT definitions that are found in the literature is provided in Table 1. It shows the diverse views of DTs across studies and domains. Table 1 also shows the key points of DTs as defined by the respective authors to facilitate the categorization of such diverse definitions and to quickly identify their similarities or differences.

**Table 1:** Definitions of digital twins (DTs) and the key points in the literature.

Definition of DT	Key points	References
A DT of a human using sensors and other data sources to create an exact virtual copy of a human being	Virtual copy	[18,27-29]
A DT is a digital replica of an object, process, or system that can be used for various purposes	Digital replica	[30-34]
A DT is an instance of the model that mimics a real patient	Mimicking or mirroring	[35-38]
A DT of a patient is a simulation of the patient’s trajectory that behaves identically to the patient in terms of outcomes	Simulation	[25,39-41]
A DT is deemed a virtual mapping of a physical entity where simulation clones the dynamic properties of physical entities in the DT	Virtual mapping	[42]
A DT is a digital representation of a physical asset reproducing its data model, behavior, and communication with other physical assets	Digital representation	[43-46]
In newer computer tomographic systems, avatars are fitted as DTs into the surface information of the positioned patients using machine learning	Avatars	[47,48]
DTs are a technology where a personalized computer model is developed that is capable of describing the physiological processes of a human body, tailored to the specific characteristics of a person	Computer model	[49]
The DT is a virtual and dynamic model in the virtual space that is completely uniform and consistent with its corresponding physical entity in the real space	Virtual model	[50,51]
The concept of DTs has emerged to enable modeling and the fusion of individual physical artifacts with digital models reflecting their status in real time	Digital model	[22,52]
DTs are virtual models that represent physical systems and their modifications in real time and there is a bidirectional flow of data, from the physical system to the virtual system and vice versa	Virtual model, bidirectional data flow	[53-56]

From Table 1, we can see that DTs are referred to as a virtual copy, digital replica, mirror, simulation, virtual map, digital representation, image, computer model, and digital and virtual models of a physical entity that are used in different ways, for example, simulating the patient trajectory, personalized treatments, monitoring real-time status, and mirroring the underlying biological systems. As can be observed, the definitions focus on different features of DTs, dependent on the application and purpose of the studies. As this domain could benefit from a universal definition and common understanding of DTs, we first discuss the known elements and underlying characteristics of DTs and then synthesize these into a definition and framework.

#### DT Architecture

In this section, we will first discuss the elements of DTs and then the characteristics of DTs that distinguish them from other similar technologies.

##### Real Entity

The aim of a DT is to create a virtual representation of physical entities which is a foundational definition of DTs [57]. In the context of manufacturing, various types of physical entities (eg, vehicles, products, components, etc) and their “real-world” existence have been studied and they are, needless to say, physical. This is a common element in the application of DTs in the field of manufacturing and health care [58]. In the health care context, the patient or hospital environment is typically considered the physical entity [2,15,18]. In ODM and CDM, operational and clinical systems, processes and workflows could also be a target of DTs, which may not be necessarily a physically existing entity. Therefore, real entity (ie, real-world entity) rather than physical entity could be a more generalized terminology.

##### Virtual Representation

A DT fully describes its real entity from the microatomic level to the macrogeometric level [59,60]. In general, a virtual representation may represent a real entity using 1 or more of the following 4 different dimensions: geometry (ie, the geometric shape, such as size), physics (ie, the physical characteristics and constraints), behavior (the dynamic behavior and responsive mechanism to internal and external changes), and rules (using historical data and domain experts to develop logical abilities, such as reasoning, evaluation, and decision-making) [61]. This representation of a real entity allows broader applications beyond static digital visualizations, for example, computer-aided design and 3D models.

##### Communication Channels

A bidirectional data connection which is one of the main components of DT systems acts as a communication channel between the real entity and its virtual counterpart. The nature of this data connection between the real entity and its virtual representation may be interpreted differently. One perspective is that the data flow between the real and virtual product is fully integrated in a way that the real product is controlled by its virtual version and a change in one of them alters the status of the other [62]. An alternative view is that the connection from the real to the virtual product is continuous in a way that the status of the real product is continuously transferred to the virtual product, while the connection of the virtual product to the real one is rather a flow of information and processes that may be applied to the actual object [63]. Regardless of the differences, this live connection is considered one of the main differentiators between DTs and traditional modeling exercises [58].

##### Computational Models

DTs use computational models that leverage gathered data to analyze, understand, monitor, and predict a system’s state and behavior. These models (eg, simulations, machine-learning implementations, and business logic) are the core of DTs [64]. Thus, a crucial step when building DTs is to create high-fidelity models that provide recommendations and capture the corresponding real entity’s geometry, physical properties, behaviors, and rules. The multimodel aspects allow the consideration of scenarios that exceed the descriptive capabilities of any single model.

##### Feedback Mechanism

DTs perform through a closed loop between real and virtual entities. This feedback mechanism is a crucial element of a DT, which could be automated, semiautomated, or human-in-the-loop decision-making depending on several factors, for example, the application, the DT’s performance, and the trust level between the DT and stakeholders. Although an automated DT is ideal, in a decision support system, especially in the context of health care where human lives are involved, the decision-making process is risk-oriented and requires a high level of frequent monitoring, consultation, and expertise. Thus, similar to other decision support algorithms, DTs may just provide suggestions and recommendations (eg, a better course of treatment) to decision makers rather than issuing orders. In this situation, the decision maker has the authority to accept or reject any offered suggestions and provide feedback to the virtual entity for further clarifications and improvements that enhance accuracy.

##### Knowledge Base

The DT is typically considered as a system that is data-driven and thrives on data. A lot of data is captured about the real entity in the development of the virtual representation. This collection of data grows as computational models are run, feedback is provided, resulting decisions are applied to the real entity, and the virtual representation is updated to represent the current state of the real entity. All of this data together constitute a knowledge base that can be used to inform future computational models and related feedback and decision-making processes.

##### User Interface

One of the advantages of DTs is their ability to handle and manage high information loads in a repetitive manner and provide feedback to decision makers. Considering the scale of their deployment, DTs may have various users (ie, stakeholders), and retrieving and displaying the necessary information for the right user may be challenging [65]. Therefore, a user interface (eg, dashboard, mobile app, etc) allows DTs to represent information in an insightful basis for decision-making and provide information to all users from varied perspectives. The dashboard may be viewed as a control room that provides users (ie, decision makers) a level of interaction to monitor the real-time state of the entities under investigation and view the results of desired experiments. In other words, users can interact with the DT through the user interface.

Considering the already explained elements, DTs have unique characteristics, as presented in Textbox 1.

**Unique characteristics of digital twins (DTs).**

- Ongoing updates of virtual representations: a continuous data connection normally is the case when Internet of Things (eg, body sensors) are the source of data collection. However, health data is normally collected from multiple sources, such as electronic health records, laboratory results, and medical images and there is a delay related to capturing those data. As the nature of this connection is to enable a DT to reflect any changes in the state of the real entity in near real time, depending on the application, the frequency of data updates in a DT may vary from seconds to hours or days.
- Close to reality: synchronization is one of the key features of a DT, which is about making sure the virtual representation presented via a DT and the actual counterpart mirror each other as closely as possible. Therefore, building a virtual representation with a high degree of similarity, particularly functional similarity, ensures that when multimodel modeling is carried out on the virtual representation, it responds in the same way we would expect the real entity to respond.
- Ability for feedback to inform decision-making: one of the important aspects of DTs is their ability to analyze and notify findings to a decision maker through an always-active feedback loop. In other words, because DTs have a full knowledge of the real entity’s historical performance and an accurate understanding of its future potential, it helps an end user to make an effective decision, for example, by considering multidimensional factors and nonlinear trade-offs that are challenging in reality.
- Multifunctionality: one of the unique aspects of the DTs is their ability to study (eg, simulate and predict) multiple physical properties and the interactions between them. This allows DTs to have many utilizations at the same time, for example, to optimize a process, continuously predict future states (eg, failures), simulate fixes and modifications to identify possible response actions, provide real-time monitoring, support the decision-making process, and provide realistic environments for virtual tests [17].

#### Our Definition and Framework

**Figure 1:**
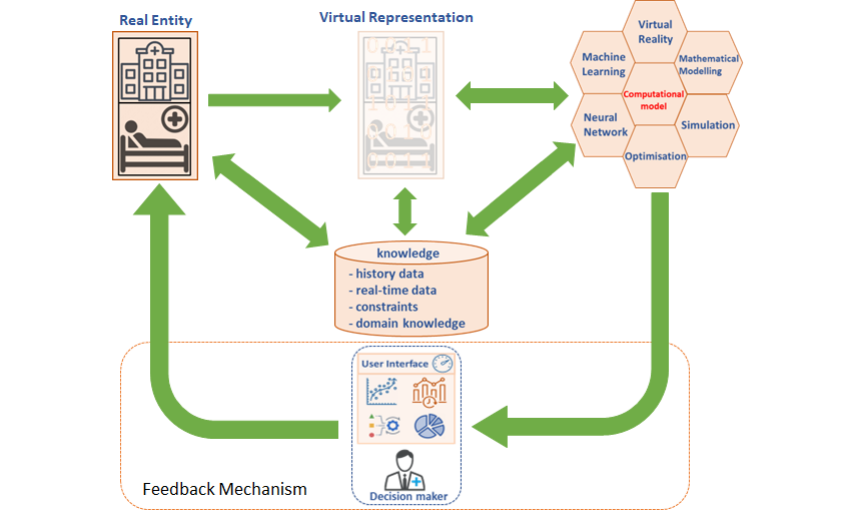
The proposed universal digital twin (DT) framework.

A universal and general definition of a DT is a knowledge-driven system that creates a close-to-reality virtual representation (twin) of a real entity (eg, clinical and operational processes). With the help of high-fidelity computational models (eg, simulation, prediction, and 3D modeling) and ongoing updates between virtual and real entities through a feedback mechanism, DTs inform decision makers (eg, administrators and clinicians) via user interfaces, to gain awareness of anticipated problems and gain an improved understanding of potential risks, challenges, and influences. The DT framework is shown in Figure 1.

In this framework, 2 environments (ie, real and virtual) are connected through data and a flow of information and processes (eg, dashboard). DTs integrate multiple approaches (eg, simulation, machine learning, and virtual reality) to create a virtual representation of the corresponding real entity. The multifunctionality of the DT provides user-care applications that are shared with the corresponding expert (eg, clinician, nurse, or administrative team) and may be transferred to the actual entity. The remainder of this paper provides a comprehensive review of the current advances and extent of DT applications to support clinical and ODM using this framework and the defined DT characteristics.

## Methods

### Guideline

We reported the review conducted in this study following the PRISMA-ScR (Preferred Reporting Items for Systematic Reviews and Meta-Analyses extension for Scoping Reviews) guidelines [66] (Multimedia Appendix 1).

### Search Strategy

The search was performed through 8 scientific databases, including PubMed, MEDLINE, Scopus, Web of Science, Embase, CINAHL, Cochrane, and gray literature databases in June 2024. As DTs for clinical and ODM within the health care domain is a very new area of research, we included most of the academic databases and gray literature databases (eg, Google Scholar and OpenGrey) with no date restrictions.

To ensure relevant studies were captured in our search process, besides digital twin*, we provided a list of search terms of which at least 1 needed to be mentioned within the title, abstract, or keywords, such as patient, hospital, Intensive Care Unit or ICU, surgery, clinic, and emergency. The following search terms were used with Boolean operators and wildcards: digital twin* AND hospital* OR *patient* OR health* OR ICU* OR ward* OR emergence* OR surger* OR ambulance* OR clinic* OR general practi* OR doctor* OR nurs*. The search strings used for all databases are shown in Multimedia Appendix 2.

### Eligibility Criteria

To identify research papers aligned with our aims, publications had to meet the following criteria: (1) they had to describe the development of a DT model for health care, (2) they had to focus on CDM or ODM in health care (excluding papers focused on medical devices), (3) they had to propose a DT model with experiments, (4) any publication date was considered, (5) only peer-reviewed original research papers were included, and (6) only articles written in English were considered. Papers proposing ideas and frameworks or describing the capabilities of DTs without applications were excluded. Papers published as literature reviews, editorials, and conference posters were excluded; duplicated papers and conference papers later published as extended journal papers were also excluded.

### Data Extraction and Data Analysis

A web-based tool for systematic or scoping reviews, called Rayyan [67], was used to conduct all stages of the screening process. We developed a standardized data extraction form in Microsoft Excel to tabulate specific information. From the included studies (ie, after performing title, abstract, and full-text screening against exclusion and inclusion criteria), the following data were extracted: the country of the first author, publication type, DT characteristics, the environment that DTs were tested within, the sample size, the service, the target area, the main underlying method used in the DTs, the type of data collected to build DTs, the *International Classification of Diseases, 10th Revision* (*ICD-10*) codes, and a summary of included studies. Most included studies are presented in the following section, while the rest are provided in Multimedia Appendix 2.

The *ICD* coding consists of 22 chapters (1 to 22), each covering specific disease areas and courses of treatment. While the papers included in our review did not explicitly provide *ICD-10* codes, we classified them based on the specific disease areas and corresponding courses of treatment mentioned in the studies. For each paper, we first identified the main focus (ie, target area) and then mapped it to the relevant *ICD-10* code, starting from the most specific level and tracing it to the highest chapter level. For example, if a study focused on prostate cancer, it would be assigned the *ICD-10* code C61 (malignant neoplasm of the prostate), which is part of chapter 2, “Neoplasms.”

In addition, in our qualitative synthesis, we systematically assessed the characteristics of DT implementations based on the developed framework, focusing on ongoing updates of virtual representations, closeness to reality, and the ability for feedback to inform decision-making. We examined the studies for discussions on the frequency and methods of data updates from real-world entities, considering various sources of data. We noted potential delays in data capture that could affect the real-time functionality of the DT. To evaluate how closely the virtual representation mirrored the actual entity, we examined the validation methods and metrics used to measure functional similarity, ensuring the DT could reliably represent real-world outcomes. We also considered the number and integration of models within each DT, as using multiple model types can enhance accuracy. We examined the mechanisms for ongoing feedback loops within the DTs, focusing on their ability to analyze historical performance and guide future decisions. This included identifying decision-support tools and user interfaces designed to help decision makers act on feedback. We also assessed whether a closed-loop system existed, where the DT actively influences decisions in the real twin. In addition, we explored how feedback is presented to various stakeholders, ensuring that insights from the DT effectively support informed decision-making in clinical and operational contexts. In assessing multifunctionality, we analyzed the range of problems the DTs were designed to address, exploring the distinct functions they served within health care. This involved evaluating the extent to which each DT tackled multiple facets of health care challenges, such as diagnosis, treatment planning, and monitoring. By adopting this structured approach, we ensured a consistent evaluation across studies, capturing the presence of these key characteristics.

Two reviewers collaborated in designing this form to capture the relevant variables effectively. Each reviewer independently extracted data from the included studies, followed by discussions to resolve any discrepancies. In instances where the reviewers could not reach an agreement, a third reviewer was consulted to extract the necessary data for those specific cases.

To synthesize the collated data, we used descriptive statistics to present frequencies and proportions for various characteristics of the included studies. Moreover, we provided a more in-depth qualitative synthesis of findings in the Results section. This qualitative synthesis highlights the nuances of the studies, such as the effectiveness of DT implementations, identified barriers and challenges, and insights into the broader implications for health care practice. The data analysis was performed using both Microsoft Excel and R software.

## Results

### Selection of Articles

**Figure 2:**
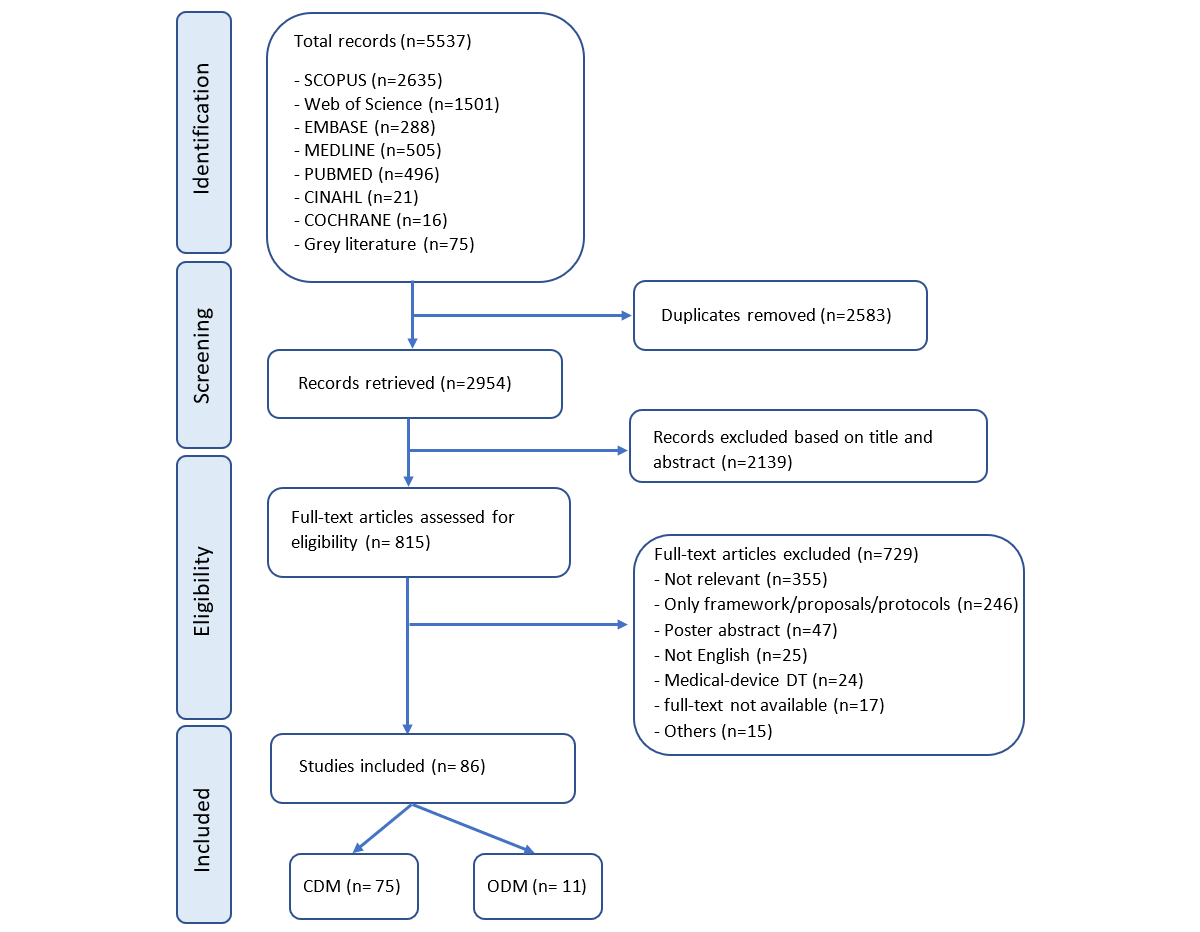
Flow diagram of the selection process of papers. CDM: clinical decision-making; DT: digital twin; ODM: operational decision-making.

A total of 5537 research papers were retrieved in the first step through all the given search databases. After screening titles and abstracts and removing duplicates, 815 (15%) papers were selected for full-text review. Considering the inclusion criteria, 86 (11%) out of 815 papers were selected for further analysis (ie, CDM: 75/86, 87% and ODM: 11/86, 13%). A total of 729 (89%) papers failed to meet the criteria. Around 34% (246/729) of these papers focused on providing ideas or frameworks and described the potential or capabilities of DTs which was the main reason for their exclusion. Moreover, 49% (355/729) of the papers were not relevant to the scope of this paper, for example, privacy and ethical aspects of DTs in health care, and big data and technology infrastructure required for DT implementations. The review process and results are shown as a PRISMA-ScR diagram in Figure 2.

### Characteristics of Included Articles

**Figure 3:**
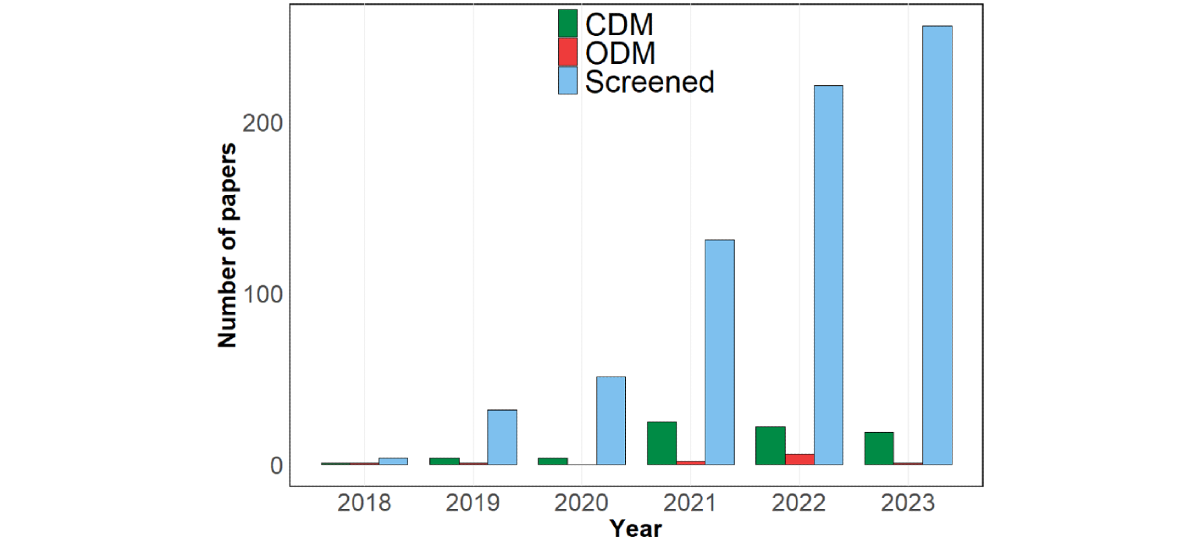
Number of papers on digital twin technology that were screened and included for the review. CDM: clinical decision-making; ODM: operational decision-making.

Papers identified through our review reflect the current upward trend of publications in the DT research area as all screened and included studies were published after 2017 as shown in Figure 3. This figure also reveals the difference in research interest among researchers between CDM (more popular) and ODM.

Analysis of the distribution of the papers by the first author’s country shows that the United States (17/86, 20%), China (11/86, 13%), and India (10/86, 12%) are the countries with the highest number of published studies. Other countries, including France, Germany, the United Kingdom, South Korea, Austria, Australia, Netherlands, Italy, and Canada contributed to DT research with at least 2 papers each. Out of the 86 papers included, 65 (76%) were published as journal papers and the rest were conference publications.

Data plays an important role within DTs as it allows the physical and virtual entities to communicate together. Besides a small number of studies (5/86, 9%) that used synthetic [40,54,68,69] or randomly generated data [50], most studies used data collected from real entities. Furthermore, analyzing the results shows that the data collected through medical images (eg, magnetic resonance imaging and computed tomography scans) are rich sources to build DT models for various purposes, for example, the His-Purkinje System [31], human vertebra [70], breast cancer [71], tibial plateau fracture [72], the tibiotalar joint [48], knee joint [52], patients with liver tumors [73,74], and the human brain [75].

For further analysis, a summary of the included papers along with their DT and health-related features is provided in Table 2. In this table, we have included characteristics that are featured in the DTs (refer to column “DT characteristics”) based on the information reported in the article.

In Table 2, the CDM-based papers were categorized based on the targeted areas of the human body using the *ICD-10* codes. Textbox 2 provides the details of all the chapters in *ICD-10*.

**Table 2:** The main features of the studied papers (N=86).

Authors		DT^a^ characteristics	Environment	Service	*ICD-10*^b^ code	Main method
**Clinical decision-making**						
	Gillette et al [31]	CTR^c^	—^d^	Optimizing treatment	Chapter 18	MM^e^
	Qi and Cao [76]	CTR	—	Efficient clinical trial	Chapter 2	Simulation
	Chakshu and Nithiarasu [45]	CTR	—	Early diagnosis	Chapter 10	NN^f^
	Doste et al [40]	CTR	—	Optimizing treatment	Chapter 9	ML^g^
	Lai et al [68]	OGU^h^, CTR, ATF^i^	Simulated	Personalize treatment	Chapter 21	RL^j^
	Tardini et al [77]	OGU, CTR, ATF	Simulated	Optimizing treatment	Chapter 2	RL
	Shamanna et al [78]	OGU, CTR, ATF	Deployed	Personalize treatment	Chapter 4	ML
	Silfvergren et al [49]	OGU, CTR, MF^k^	Deployed	Early diagnosis	Chapter 21	MM
	Shamanna et al [79]	OGU, CTR, ATF	Deployed	Personalize treatment	Chapter 4	ML
	Hwang et al [80]	CTR	—	Optimizing treatment	Chapter 11	3D models
	Lal et al [81]	CTR	—	Early prevention	Chapter 1	Simulation
	Cho et al [82]	CTR	—	Knowledge sharing	Chapter 19	3D models
	Shamanna et al [83]	OGU, CTR, ATF	—	Personalize treatment	Chapter 4	ML
	Chakshu at al [54]	CTR	—	Early diagnosis	Chapter 9	NN
	Ahmadian et al [70]	CTR	—	Early prevention	Chapter 19	NN
	Golse et al [84]	OGU, CTR, ATF	Simulated	Early prevention	Chapter 11	MM
	Ahmadian et al [85]	CTR, MF	—	Early prevention	Chapter 19	NN
	Wu et al [71]	CTR	—	Personalize treatment	Chapter 2	MM
	Talukder et al [86]	CTR	—	Accurate prognosis	Chapter 221	ML
	Batch et al [87]	CTR	—	Personalize treatment	Chapter 2	NN
	Aubert et al [72]	CTR	—	Optimizing treatment	Chapter 19	Simulation
	Hernigou et al [48]	CTR	—	Personalize treatment	Chapter 19	NN
	Tai et al [88]	OGU, CTR, ATF	Deployed	Optimizing treatment	Chapter 1	NN
	Palaniappan and Surendran [55]	CTR	—	Early prevention	Chapter 11	NN
	Chen [52]	OGU, CTR, ATF	Deployed	Early prevention	Chapter 19	MM
	Baena et al [89]	CTR	—	Early prevention	Chapter 13	Simulation
	Barbiero et al [35]	OGU, ATF	Simulated	Knowledge sharing	Chapter 21	NN
	Roy et al [90]	OGU	Simulated	Early prevention	Chapter 9	MM
	Allen et al [39]	OGU	Simulated	Early prevention	Chapter 9	NN
	Hussain et al [91]	—		Early prevention	Chapter 9	ML
	Meraghni et al [32]	OGU	Simulated	Early diagnosis	Chapter 2	MM
	Scheuermann et al [92]	OGU, CTR, ATF	Deployed	Early prevention	Chapter 21	ML
	Semakova et al [93]	—	—	Early prevention	Chapter 9	MM
	Ossai and Wickramasinghe [94]	—	—	Early prevention	Chapter 20	BN^l^
	Martinez-Velazquez et al [27]	—	—	Early prevention	Chapter 9	NN
	Chakshu et al [56]	—	—	Early diagnosis	Chapter 9	MM
	Shi et al [73]	OGU, CTR, ATF	Deployed	Optimizing treatment	Chapter 2	MM
	Kim et al [95]	—	—	Early diagnosis	Chapter 2	ML
	Manocha et al [38]	OGU	Simulated	Real-time monitoring	Chapter 21	NN
	Venkatapurapu et al [36]	CTR	—	Optimizing treatment	Chapter 11	Mechanistic model
	Wu et al [69]	OGU, CTR	Simulated	Early diagnosis	Chapter 27	MM
	Zhou et al [96]	CTR	—	Personalize treatment	Chapter 21	MM
	Lauzeral et al [74]	CTR	—	Personalize treatment	Chapter 1	MM
	Wan et al [75]	CTR	—	Accurate prognosis	Chapter 6	ML
	Hernigou et al [51]	CTR	—	Knowledge sharing	Chapter 19	3D models
	Azzolin et al [97]	OGU, CTR	Simulated	Optimizing treatment	Chapter 9	MM
	Ang et al [98]	CTR	—	Optimizing treatment	Chapter 21	MM
	van Osta et al [99]	CTR	—	Early diagnosis	Chapter 9	MM
	Kardampiki et al [100]	CTR	—	Optimizing treatment	Chapter 9	MM
	Jiang et al [101]	OGU, CTR, ATF	Deployed	Real-time monitoring	Chapter 21	Optimization
	Kobayashi et al [102]	OGU, CTR, ATF	Deployed	Real-time monitoring	Chapter 5	NLP^m^
	Förster et al [103]	CTR	—	Personalize treatment	Chapter 21	Simulation
	Fu et al [104]	CTR	—	Personalize treatment	Chapter 28	NN
	Gillette et al [105]	CTR	—	Knowledge sharing	Chapter 28	MM
	Goodwin et al [106]	OGU, CTR, ATF	Deployed	Optimizing treatment	Chapter 4	Optimization
	Yuan et al [107]	—	—	Early diagnosis	Chapter 21	ML
	Alcaraz et al [108]	OGU, CTR	Deployed	Optimizing treatment	Chapter 28	MM
	Shu et al [109]	OGU, ATF	Deployed	Optimizing treatment	Chapter 19	Simulation
	Sarp et al [110]	CTR, MF	—	Real-time monitoring	Chapter 19	NN
	Demir et al [111]	CTR	—	Personalize treatment	Chapter 11	MM
	Koopsen et al [112]	CTR	—	Optimizing treatment	Chapter 21	MM
	Batagov et al [113]	CTR	—	Early diagnosis	Chapter 4	ML
	Joshi et al [114]	OGU, CTR, ATF	Deployed	Personalize treatment	Chapter 4	ML
	Grieb et al [115]	CTR	—	Early diagnosis	Chapter 2	Graph-based network
	Zhang et al [116]	CTR, MF	—	Optimizing treatment	Chapter 4	ML
	Rouhollahi [117]	CTR	—	Early prevention	Chapter 17	NN
	Serra et al [118]	CTR	—	Early prevention	Chapter 9	Simulation
	Chahal [119]	CTR	—	Early diagnosis	Chapter 4	NN
	Uyttendaele et al [120]	OGU, CTR, ATF	Deployed	Optimizing treatment	Chapter 28	3D models
	Ložek et al [121]	CTR	—	Optimizing treatment	Chapter 9	MM
	Thamotharan et al [122]	OGU, CTR, ATF	Deployed	Optimizing treatment	Chapter 4	NN
	Cappon et al [123]	CTR	—	Personalize treatment	Chapter 4	Simulation
	Salvador et al [124]	CTR, ATF	—	Personalize treatment	Chapter 28	MM
	Dubs et al [125]	CTR, ATF	—	Optimizing treatment	Chapter 9	MM
	Khan et al [126]	OGU, CTR, ATF	—	Real-time monitoring	Chapter 28	ML
**Operational decision-making**						
	Karakra et al [50]	OGU, ATF	Simulated	Real-time monitoring	—	Simulation
	Augusto et al [127]	CTR	—	Facility management	—	Simulation
	Pilati et al [53]	OGU, CTR, ATF	Deployed	Efficiency improvement	—	Simulation
	Maïzi and Bendavid [128]	OGU	Simulated	Efficiency improvement	—	Simulation
	Karakra et al [129]	OGU, ATF	Simulated	Real-time monitoring	—	Simulation
	Possik et al [30]	—	—	Efficiency improvement	—	Simulation
	Chen et al [42]	—	—	Knowledge sharing	—	Simulation
	Zhong et al [41]	CTR, ATF, MF	—	Medical resource allocation	—	Simulation
	Ritzinger et al [37]	OGU, CTR, ATF	Simulated	Medical resource allocation	—	Optimization
	Basaglia et al [130]	CTR	—	Facility management	—	Simulation
	Zackoff et al [131]	CTR	—	Facility management	—	3D models

^a^DT: digital twin.

^b^ICD: International Classification of Diseases, 10th Revision.

^c^CTR: close to reality.

^d^Not available.

^e^MM: mathematical modeling.

^f^NN: neural network.

^g^ML: machine learning.

^h^OGU: on-going updates.

^i^ATF: ability to feedback.

^j^RL: reinforcement learning.

^k^MF: multifunctional.

^l^BN: Bayesian network.

^m^NLP: natural language programming.

Studies included in this review covered more than half (14) of all 22 chapters. Chapter 19 (“Diseases of the circulatory system”; 14/86, 16%) and chapter 21 (“Factors influencing health status and contact with health services”; 12/86, 14%) include the most studies, followed by chapter 4 (“Endocrine, nutritional, and metabolic diseases”; 10/86, 12%). These findings demonstrate the diversity of health care areas where DT models have been applied.

In terms of health care services in the included studies, for CDM, optimizing treatment (20/86, 23%), early prevention (16/86, 19%), and personalized treatment (15/86, 17%) are the 3 main service areas studied in the included papers, while for ODM, facility management (3/86, 3%) and efficiency improvement (3/86, 3%) are the most popular areas.

Regarding the main computational approach used in the proposed DTs, mathematical modeling (24/86, 28%), for example, moving least square and physiological models is the most common method followed by simulation techniques, for example, discrete event simulation (17/86, 20%) and neural network-based algorithms, for example, deep convolutional neural networks and graph neural networks, (17/86, 20%). Unsurprisingly, simulation-based models, such as discrete event simulation (9/11, 81%) are the most popular method used for ODM (as they can allow decision makers to investigate “what if” scenarios by quantifying the impact of potential changes in the operational settings [132]. The detailed distribution of disease areas, services, and computational methods in the included papers are provided in the Multimedia Appendix 3.

The following was also observed for these included studies: out of 86, only 32 (37%) studies demonstrated ongoing updates of virtual representations in their developed system, although it is the most fundamental characteristic of a DT. Among those 32 studies, 16 (50%) tested the developed system in a simulated environment, and the remainder were tested in real environments. Of the 4 defined characteristics of DTs, multifunctionality is a characteristic that was found to be lacking in most of the studies (81/86, 94%). Among the included papers, all studies missed at least one of the DT’s characteristics, and therefore, did not materialize all 4 characteristics of DT.

Although none of the studies had all the desired DT characteristics, the reported impact of the deployed DTs offers promising insight into their effectiveness. For example, a DT-based program for patients with type 2 diabetes helped all 12 insulin-dependent patients who attended the program to stop insulin injections and helped 38 out of 56 to stop taking metformin [79]. A DT technology used for assisting a surgeon with real-time performance of thermal ablation (a treatment to destroy tumor cells) provided significantly better results compared to other existing methods [73]. A DT for estimating the risk of posthepatectomy liver failure (a leading cause of postoperative death) was developed by modeling blood circulation and estimating 2 important determinants of the disease that could not be predicted before the development of the DT [84].

**Chapters in the International Classification of Diseases, 10th Revision.**

- Chapter 1: Certain infectious and parasitic diseases
- Chapter 2: Neoplasms
- Chapter 3: Diseases of the blood and blood-forming organs and certain disorders involving the immune mechanism
- Chapter 4: Endocrine, nutritional and metabolic diseases
- Chapter 5: Mental and behavioural disorders
- Chapter 6: Diseases of the nervous system
- Chapter 7: Diseases of the eye and adnexa
- Chapter 8: Diseases of the ear and mastoid process
- Chapter 9: Diseases of the circulatory system
- Chapter 10: Diseases of the respiratory system
- Chapter 1: Diseases of the digestive system
- Chapter 12: Diseases of the skin and subcutaneous tissue
- Chapter 13: Diseases of the musculoskeletal system and connective tissue
- Chapter 14: Diseases of the genitourinary system
- Chapter 15: Pregnancy, childbirth and the puerperium
- Chapter 16: Certain conditions originating in the perinatal period
- Chapter 17: Congenital malformations, deformations and chromosomal abnormalities
- Chapter 18: Symptoms, signs and abnormal clinical and laboratory findings, not elsewhere classified
- Chapter 19: Injury, poisoning and certain other consequences of external causes
- Chapter 20: External causes of morbidity and mortality
- Chapter 21: Factors influencing health status and contact with health services
- Chapter 22: Codes for special purposes

## Discussion

### Principal Findings

The main finding of this study is that while DT systems in health care have shown promising results across multiple areas, they still do not fully incorporate all the desirable characteristics of a comprehensive DT. Nonetheless, DTs are making significant advances in clinical and ODM processes, particularly in areas such as cardiovascular disease, diabetes, cancer, orthopedics, and emergency department operations. The ability to integrate multiple techniques and technologies, such as big data, cloud computing, communication, virtual reality, blockchain, IoT, simulation, prediction, and optimization helps to create a digitally enabled environment for DTs to encapsulate and provide solutions for complex and multidisciplinary problems that were difficult to deal with using traditional methods. Key examples of such solutions include real-time monitoring, treatment optimization, risk factor early intervention, efficient clinical trial design, and improving operational efficiency.

The results of this study showed that the development and deployment of DTs within health care settings still lack maturity. Our review of the literature revealed a lack of a common definition and some inconsistency in the use of the term *digital twin*. Consequently, the focus of this study was shifted toward defining a DT and its underlying elements to support a common understanding and suggest a framework for designing and developing DTs. Building a DT is a complex process in terms of modeling, functionality, domain specificity, and data connectivity process. Therefore, research articles on DTs need to be transparent about their purpose (ie, functionality), the virtual representation that is being created, the data (eg, the frequency of data update, what data elements need to pass from the real entity to the virtual representation and vice versa), the feedback mechanism (eg, what are the outputs, who is receiving that feedback information, etc), and the computational models used (eg, the accuracy of the models and justification for the model's selection). This transparency helps any developed DTs be understandable and their potential for translation be gauged.

Assessing the identified studies against the defined characteristics of DTs revealed that while there are emerging applications of DTs for CDM and ODM, current efforts were not mature enough to include all desired features. As shown in Table 2, most of the reviewed studies did not include ongoing updates of virtual representations in their developed systems. Although they may not materialize a major characteristic of a DT, that is, fed by live data and synchronized with real-life events, they are proof-of-concept studies that provide evidence for the appetite for DTs in health care. One of the main aspects of DTs that is lacking in the included studies is the multifunctionality and scale of the proposed DTs. Studying the input and output data of the developed DTs revealed that they are mostly designed with a single purpose although DTs are designed to create a complete virtual description of a real entity that is accurate at both the micro and macrolevels. The size of the samples used in the included studies shows that the developed DTs are still in experimental stages, and to fully embrace DT technologies, they need to move from custom expert-driven implementations to accessible robust implementations at scale [133].

As DTs are an emerging research area, especially in health care, it is not surprising to witness misconceptions, misunderstandings, and misrepresentations. It has been discussed that DTs are closely related to other research areas, such as 2D or 3D modeling, system simulation, and digital prototyping and this is a big factor in this confusion [2,26]. Reviewing some of the definitions of DTs over the years provides insight into their growth and development. Considering the fact that researchers are still trying to adopt DT concepts and technologies from manufacturing to health care and figure out how DTs can be implemented in such complex systems, it is understandable that there are still a few steps to go in designing, building, and executing a true DT system within a health care setting. Reviewing the selected papers in this study revealed that DT applications in health care are still in a transformative stage moving from offline systems, for example, digital models and simulations without any real-time connection with the real entity, toward DTs encompassing the aforementioned characteristics, and there is improved awareness of what separates DTs from similar technologies as interest in this field grows.

While none of the reviewed studies possesses all desired DT characteristics, the current state of the deployed models suggests that DTs have the potential for substantial impact once fully developed. They also showcase the versatility of DTs in CDM and ODM applications, from screening, diagnosing, detecting disease, and personal treatment to clinical trial design and optimizing hospital operations. While interest in the applications of DTs in health care is evident, the key focus area might be in understanding how health care operations could benefit from DT use. Developing DTs for designing clinical trials exemplifies how they provide personalized health care by allowing patient-specific responses to therapies to be simulated and treatment plans to be optimized [76]. Nevertheless, the impact of DTs can be extended to population health by providing insights into disease patterns and treatment efficacy across varied populations. Economically, they reduce costs related to traditional clinical trials, such as participant recruitment and logistics, and also position organizations at the forefront of innovation, attracting investment and fostering industry growth. Socially, virtual testing minimizes risks to human participants and enhances trial transparency, as well as addressing disparities by generating inclusive data that accurately represents diverse demographic groups. Thus, DTs promise significant advancements, offering broader societal and economic benefits.

### Challenges and Barriers to Implementing DTs in Health Care

#### Overview

Alongside the promising opportunities, the application of DTs faces several challenges and concerns that could impede their full potential. As with other systems involving the exchange of health information, data security and integrity are essential for preserving patient privacy and ensuring high-fidelity models can be created. To implement DTs widely, significant investments are needed in both digital and physical infrastructure, including scalable cloud computing and high-performance computing for storing and processing the vast amounts of generated data and running complex simulations, and IoT devices with reliable networking for real-time data gathering and supporting rapid data transfer. Such a system upgrade will be particularly challenging due to the need to integrate DTs with existing legacy systems that often lack standardization and interoperability. To facilitate this transformation, it is essential to mitigate these challenges by establishing standardized data formats, communication protocols, and data exchange mechanisms that enhance efficient information flow across systems [134]. Besides, DTs’ validation should be first established for an end user to trust the feedback, similar to how predictive performance and clinical validation of artificial intelligence and machine learning models are assessed before use.

There are some other unique aspects related to health care that make the implementation of a DT model challenging but compelling. One important property of DTs is having real time, live, and continuous data flow. However, health data normally is sourced from multiple collections, such as electronic health records, laboratory results, and medical images and there is a delay related to capturing those data. For example, the results of blood tests may take up to 24 hours to be ready or care providers may update coding within a patient record at the end of their stay in the hospital. Capturing accurate data is, in fact, another challenge. Today, the health care industry contributes approximately 30% of the data worldwide and by 2025, it is expected to reach close to 5000 digital device interactions per person per day [135]. This deluge provides an opportunity for developing data-driven models, such as DTs; however, assembling these data from various sources which are normally stored in different formats remains a significant challenge, along with the accuracy of underlying patient-level data stored within datasets. Moreover, the collection and storage of extensive data raises significant data privacy and security concerns, particularly given the sensitive nature of patient information. Solutions involve implementing robust security measures like encryption and access controls to ensure the protection of data during sharing and analysis [18,25,134,136].

Another big difference in DT applications in health care compared to other industries is human involvement, which raises a myriad of ethical issues. It has been discussed that a DT based on a virtual representation of a human may be created in such a way that the virtual version acts on behalf of the actual person and directly affects the person [137]. Therefore, not only does the digital model need informed consent, but also the person needs to be granted adequate control over their digitally-twinned representation. Trust is another important consideration along the path of the development of DTs in health care. Because of the high level of sensitivity working with health data, DTs as an emerging technology not only need reliable results but also the trust of health care providers.

#### Future Directions and Policy Considerations for DTs in Health Care

With the rapid growth of DT models, driven by global investments in underlying technologies, such as IoT, and their potential to outperform traditional methods [138], the application of DTs in clinical and ODM is poised to accelerate in the foreseeable future. Effective policy improvement, such as enforcing interoperability standards, providing funding and incentives for technological upgrades, updating data privacy laws, and establishing clear artificial intelligence and machine learning regulatory frameworks will be crucial in facilitating this transmission.

### Limitations and Strengths

This study has several limitations. First, the scoping review was limited only to papers published in English. Second, the search process was restricted only to original papers with the term *digital twin* included in either the title, abstract, or keywords which may have resulted in excluding other closely relevant studies that may not have used this term. Third, in the inclusion criteria, we focused on DT applications within ODM and CDM; however, this may have led to excluding a portion of DT applications in other health domains, such as medicine and drug discovery. Fourth, this study reviewed a wide range of DT applications in health care delivery without critically assessing their reporting quality. Therefore, more detailed quantitative analyses can be considered as future research activities that are beyond the scope of this work. Fifth, while our search strategy was comprehensive, some relevant studies indexed under different terminology or metadata, such as MeSH terms, may have been unintentionally missed. Finally, there are articles published between the completion of the search process (June 2024) and the publication of this study that we were not aware of and therefore were not included in this review.

Despite the challenges and limitations highlighted earlier, it is important to acknowledge the strengths of our work. This study offers a comprehensive framework for defining the key characteristics of DTs in health care, providing an essential resource for future research and practical applications. Our review not only outlines the current advancements in DT technology but also pinpoints critical gaps between present capabilities and their full potential, especially in clinical and ODM. The proposed framework serves as a guide for researchers and health care providers in developing DT systems, offering a reference for incorporating all the desirable characteristics of a comprehensive DT to effectively implement and use these technologies. Considering the rapid development of DTs, our review is both timely and highly relevant for researchers and health care practitioners. We adopted a deliberately broad scope, encompassing studies from diverse clinical settings and health care disciplines to ensure a comprehensive understanding of DT applications. By incorporating both academic and gray literature, our findings are further strengthened, offering a solid basis for those working to develop and implement DT systems.

### Conclusions

The application of DTs in health care is exponentially growing and it is expected to play a major role in decision-making processes at both operational and clinical levels. This scoping review presents a broad overview of currently developed DT technologies, and the key findings can be summarized as follows: before this study, a common definition of DTs was missing, and researchers often confused other approaches with DTs. In this study, we provided a detailed description of DT elements and characteristics and synthesized these into a universal definition and architecture for DTs for clinical and operational decision support. Second, most prior work has not materialized a major characteristic of DTs: although bidirectional data connection between a real entity and the corresponding virtual one is a fundamental element of a DT, the ongoing update characteristic is lacking in most of the included papers. Third, the implementation of DTs is still in its infancy. Only 19% of the included studies tested the developed DT in a real environment and 95% of them were designed with a single-purpose functionality. Further study is warranted to explore the potential of DTs, for example, integrating ongoing updates into DT models, evaluating practical implementations in health care settings (ie, assessing effectiveness, feasibility, and scalability), and enhancing versatility for broader functionality and seamless integration.

## Appendix

Multimedia Appendix 1 PRISMA-ScR (Preferred Reporting Items for Systematic Reviews and Meta-Analyses extension for Scoping Reviews) checklist.

Multimedia Appendix 2 Raw data from the review.

Multimedia Appendix 3 Distribution of disease areas, services, and computational methods in the included papers.

## Abbreviations

CDM: clinical decision-making
DT: digital twin
ICD-10: International Classification of Diseases, 10th Revision
IoT: Internet of Things
ODM: operational decision-making
PRISMA-ScR: Preferred Reporting Items for Systematic Reviews and Meta-Analyses extension for Scoping Reviews
