# Supplementary material for: Digital Twins for Clinical and Operational Decision-Making: Scoping Review
Source: J Med Internet Res. 2025 Jan 8;27:e55015. doi: 10.2196/55015 (PMC11754991; doi:10.2196/55015)
Supplement: Multimedia Appendix 2 [file jmir_v27i1e55015_app2.docx]

Review search strings used for each database

| Scopus | ( TITLE-ABS-KEY ( "digital twin" ) AND TITLE-ABS-KEY ( "hospital*" OR "*patient*" OR "health*" OR "ICU*" OR "ward*" OR "emergency*" OR "surger*" OR "ambulance*" OR "clinic*" OR "general practi*" OR "doctor*" OR "nurs*") ) AND ( LIMIT-TO ( LANGUAGE , "English" ) ) |
| --- | --- |
| PubMed | ("digital twin*"[Title/Abstract] AND ("hospital*"[Title/Abstract] OR "patient*"[Title/Abstract] OR "health*"[Title/Abstract] OR "icu"[Title/Abstract] OR "ward*"[Title/Abstract] OR "emergency*"[Title/Abstract] OR "surger*"[Title/Abstract] OR "ambulance*"[Title/Abstract] OR "clinic*"[Title/Abstract] OR "general practi*"[Title/Abstract] OR "doctor*"[Title/Abstract] OR "nurs*"[Title/Abstract])) AND (english[Filter]) |
| Web of Science | TS=("digital twin*" AND ("hospital*" OR "*patient*" OR "health*" OR "ICU*" OR "ward*" OR "emergency*" OR "surger*" OR "ambulance*" OR "clinic*" OR "general practic*" OR "doctor*" OR “nurs*”)) |
| Medline | TS=("digital twin*" AND ("hospital*" OR "*patient*" OR "health*" OR "ICU*" OR "ward*" OR "emergency*" OR "surger*" OR "ambulance*" OR "clinic*" OR "general practic*" OR "doctor*" OR “nurs*”)) |
| Embase | 'digital twin*' AND ('hospital*':ti,ab,kw OR 'patient*':ti,ab,kw OR 'health*':ti,ab,kw OR 'icu*':ti,ab,kw OR 'ward*':ti,ab,kw OR 'emergency*':ti,ab,kw OR 'surger*':ti,ab,kw OR 'ambulance*':ti,ab,kw OR 'clinic*':ti,ab,kw OR 'general practic*':ti,ab,kw OR 'doctor*':ti,ab,kw OR nurs*':ti,ab,kw) |
| CINAHL | AB "digital twin*" AND AB ( "hospital*" OR "*patient*" OR "health*" OR "ICU*" OR "ward*" OR "emergency*" OR "surger*" OR "ambulance*" OR "clinic*" OR "general practic*" OR "doctor*" OR “nurs*”) |
| Cochrane | "digital twin" in Title Abstract Keyword AND "hospital*" OR "*patient*" OR "health*" OR "ICU*" OR "ward*" OR "emergency*" OR "surger*" OR "ambulance*" OR "clinic*" OR "general practic*" OR "doctor*" OR "nurs*" in Title Abstract Keyword |
| Google scholar | allintitle: "digital twin" AND "hospital*" OR "*patient*" OR "health*" OR "ICU*" OR "ward*" OR "emergency*" OR "surger*" OR "ambulance*" OR "clinic*" OR "general practic*" OR "doctor*" OR “nurs*” |

Bibliometric information and methodological characteristics of reviewed studies on digital twin

| Reference | First author country | Real entity | Publication type | Method | N (sample size) | Type of data | Summary | Funding sources |
| --- | --- | --- | --- | --- | --- | --- | --- | --- |
| [1] | Austria | His-Purkinje System within the ventricles | Journal | His–Purkinje system (HPS) | 1 patient | MRI data | proposing a 2-stage method for generating the His–Purkinje system (HPS) to model ventricular conduction systems | University |
| [2] | USA | patients with non-small cell lung cancer (NSCLC) | Journal | Simulation | 25,708 records from 524 patients | patient data, e.g., lesion diameter measures | Virtual trial simulation to explore the feasibility of treatment beyond progression with pembrolizumab in patients with PD-L1 TPS ≥50%. Virtual trials predicted progression-free survival (PFS) from pembrolizumab beyond progression to be comparable with salvage chemotherapy in patients whose PD was due to nontarget progression. |  |
| [3] | UK | patients with pneumonia | Journal | Neural network algorithms | >1895 patients | patient data, e.g., age, gender, heart rate, pulse, systolic and diastolic blood pressures, respiration rate, and spot oxygen saturation | They used deep learning methods to build digital twin models to identify and prioritise critical cases amongst severe pneumonia patients by proposing strategies to generate severity indices. The severity indices are the probability of death and the probability of requiring mechanical ventilation. | government |
| [4] | UK | ventricle | Journal | Support vector machines (SVM) model | 2496 synthetic records and 365 patients | clinical data: heart meshes, cellular and tissue electrical properties. | To determine the site of origin (SOO) in outflow tract ventricular arrhythmias (OTVAs), i.e., the differentiation between left and right ventricular origin, before an ablation procedure, they proposed the use of detailed electrophysiological simulations of OTVAs to train the support vector machines (SVM) classification algorithm to predict the ventricular origin of the SOO of ectopic beats. They showed that the model can predict the SOO with an accuracy of 0.86 in the clinical database of 334 patients, and 0.84 in the second clinical database of 31 patients. | University |
| [5] | China | heart | conference | reinforcement learning algorithm | 3 virtual patients | heart-related data: electrograms (EGM), electrocardiograms (ECG) and signals sensed by an ICD | They focused on implantable cardioverter defibrillators (ICDs) with the goal of finding individualised device parameter settings. To that end, they proposed a reinforcement learning framework using ECG signals from ECG/Holter patches to infer the patient’s physiological states using virtual heart models. | Not supplied |
| [6] | USA | therapy process for patients with head and neck cancer | Journal | Deep Q-learning (DQL) | 536 | clinical and demographic data: age, gender, smoking status, primary tumour, lymph nodes, tumour laterality, tumour subsite, Prescribed chemotherapy, Chemotherapy modification type | They proposed a deep Q-learning (DQL) algorithm to dynamically select treatment based on multiple clinically relevant outcomes from data specific to patients with head and neck cancer. The proposed DQL tries to find the best sequential decision-making makings such as the following treatment after receiving induction chemotherapy (IC) considering a reduction in tumour size, time of local region control, and survival time | non-government |
| [7] | India | patients metabolism | Journal | Machine learning algorithm | 463 | glucose profiles, weight,, blood pressure, food intake, sleep pattern | they developed a DT model called Twin Precision Nutrition (TPN) that understands the patients' unique metabolic impairment and provides daily precision nutrition guidance to the patient. Using AI technologies, they collect the corresponding data which is then analysed by ML algorithms to predict the glucose response to specific foods. Using this information, they are able to provide specific daily food recommendations to skip glucose spikes. |  |
| [8] | Swiden | metabolic function | Journal | Mathematical modelling methodology | 3 | clinical data e.g., carbohydrate amount, protein amount, insulin fluxes, sex, height and weight. | they proposed a DT model that predicts the effect of different diets and fasting schedules and also quantifies glycogen levels and metabolic control. They extend some of the existing models by adding new features such as intracellular metabolism in the liver, long-term energy regulation via the new states for liver and kidney glycogen, protein metabolism, and hepatic interconversion between glucose and amino acids. | government |
| [9] | India | patients metabolism | Journal | Machine learning algorithm | 64 | glucose profiles, weight, BMI, duration of diabetes, blood pressure, food intake, sleep pattern | they developed a DT model called Twin Precision Nutrition (TPN) that uses continuous glucose monitor (CGM), Internet of Thing (IoT) technologies, mobile apps, and ML methods to provide food to patients with type 2 diabetes that helps them avoid the glucose spikes. they measured the algorithm by monitoring the changes in haemoglobin A1c (HbA1c), as the primary endpoint during the 90 days of study. They reported that 9.4% of patients had an HbA1c < 6.5% at baseline which increased to 24.6% at 30 days, 38.0% at 60 days, and 39.7% at 90 days. |  |
| [10] | South Korea | human mouth | Journal | CAD software | 1 | intraoral and facial scan data, eccentric and opening movements | After gathering related data such as intraoral and facial scan information, CBCT information, and jaw movement data, they created a digital model for the full-mouth rehabilitation of a patient. They used the simulation model to accurately position the crown at the location of the diagnostic tooth arrangement and also to minimise the occlusal adjustment in the patient's mouth. | government |
| [11] | USA | patients with sepsis | Journal | simulation models, Bayesian networks | 29 | demographic, clinical and laboratory data | They developed a DT model that simulates critically ill patients and then predicts the response of sepsis patients to any specific treatments during the first 24 hours. Using Bayesian networks, they used a directed acyclic graph to define the causal relationship among organ systems and specific treatments. |  |
| [12] | South Korea | adult jaws | Journal | FaceGide program | 50 | facial scans and cone-beam computed tomography (CBCT) images | They developed a DT model of the Korean female facials by using facial scans and cone-beam CT scans to investigate the sagittal relationship between the forehead and the maxillary central incisors before and after orthodontic treatment. They showed that Korean females seeking orthodontic treatment had their maxillary central incisor anterior to the glabella | government |
| [13] | India | patients metabolism | Journal | Machine learning algorithm | 64 | glucose profiles, weight, BMI, duration of diabetes, blood pressure, food intake, sleep pattern | Same as "Reducing HbA1c in Type 2 Diabetes Using Digital Twin Technology-Enabled Precision Nutrition: A Retrospective Analysis" with a more detailed example |  |
| [14] | UK | Blood pressure waveforms | Journal | inverse analysis, neural networks | 8516 virtual records | age, weight, height, pulse pressure, cardiac output, pulse wave velocity | they developed a cardiovascular Digital Twins (CDT) using a generated synthetic dataset for the detection and diagnosis of AAA. The CDT is implemented using inverse analysis to detect the systematic blood flow in the cardiovascular system by reversely calculating blood pressure waveform in various blood vessels of the body from pressure waveform from three non-invasively accessible vessels (carotid, femoral, and brachial) and then using neural networks to predict the AAA and its severity. They showed that this method can provide 99.91% accuracy in detecting AAA. | University |
| [15] | USA | human vertebra | Journal | deep convolutional generative adversarial network | 1 | imaging data of the trabecular bone | they proposed a framework called ReconGAN as a digital twin of the human vertebra which enables to prediction of the risk of vertebra fraction (VF). The ReconGAN relies on a uses a 3D deep convolutional generative adversarial network (DCGAN) trained with grayscale images of the trabecular bone tissue obtained via micro-QCT from cadaveric samples to build geometrical models of whole vertebra. | University |
| [16] | France | hemodynamic systems | Journal | A mathematical (0D) model | 47 | heart rate, cardiac output, portal flow, hepatic artery flow, and mean arterial pressure, among many | They developed a DT model of the liver to predict the risk of post-hepatectomy portal hypertension which is a major cause of liver failure. To that end, they developed a mathematical 0D model that simulates the entire blood circulation to reflect preoperative conditions and uses the measured data of each patient as the model variables |  |
| [17] | USA | human vertebra | Journal | Deep Convolutional Generative Adversarial Network | - | micro-QCT imaging data | This paper follows up their previous work; "Toward an artificial intelligence-assisted framework for reconstructing the digital twin of the vertebra and predicting its fracture response". In this work, they developed a model to predict the fracture response after VP procedures. They used a CFD-based model to simulate the injected cement morphology which helps to reconstruct a more realistic model of vertebra. | University |
| [18] | USA | patients with Triple-Negative Breast Cancer | Journal | mathematical modeling | 56 | MRI data | They developed a DT model to predict breast cancer patient response to neoadjuvant systemic therapy (NAST). To that end, they integrate MRI data and biologically based mathematical modelling and show that the proposed model can accurately predict tumour status, and whether further dosing with doxorubicin/cyclophosphamide (A/C) should be continued, or if an alternative intervention should be considered. | Institutional |
| [19] | India | disease knowledge | Journal | node2vec (an unsupervised learning algorithm) | - | ontologies data and patient data from EHR | They develop a graph-based model that helps healthcare workers provide accurate diagnoses by integrating comprehensive disease-related knowledge such as Disease Ontology, disease symptoms, SNOMED CT, DisGeNET, and PharmGKB data. The model uses a node2Vec algorithm and builds a knowledge graph with multiple types of nodes (e.g., disease nodes, genes, ICD10) and relationships. | no funding |
| [20] | USA | cancer | Journal | Neural network algorithms | >14,000 reports of >1,500 patients | Radiology report | they developed a model with the aim of improving the detection of metastatic disease over time using over 700,000 structured radiology reports. They proposed three neural network-based algorithms (e.g., convolutional NN and recurrent NN)focusing on three separate organs (e.i., lung, liver and adrenal). To build the models, they consider multiple consecutive reports of individuals throughout the patient's treatment history and show that NN models can extract cancer progression patterns and provide higher performance compared to single-report-based prediction ones. |  |
| [21] | France | tibial plateau fracture | Journal | Finite element analysis | 1 | 3D X-Ray images | They built a patient-specific element finite model approach for patients suffering from tibial plateau fractions using postoperative 3D X-ray images. They discuss that this model helps to simulate different possible interventions and investigate the mechanical strength and stress distributions in the bones and therefore improve postoperative management decision-making. | Government, Institutional |
| [22] | France | the tibiotalar joint | Journal | Deep learning algorithm | 5 | CT scan images | The authors developed 3D models of distal extremities employing ML techniques and the use of CT scan images to identify the motion axis of the tibiotalar joint. They discuss how this model can be helpful for a better understanding of ankle surgery particularly total ankle arthroplasty. They first transferred the CT images to Stereo-Lithography (STL) file format and then used CAD Catia software to build the 3D model. | Not supplied |
| [23] | China | patients with lung cancer | Journal | a robust auxiliary classifier generative adversarial network (rAC-GAN) | 1462 |  | They developed a DT model to predict lung cancer with pulmonary embolism (PE)-positive with over 90% accuracy. They developed a deep learning algorithm model that uses patient data such as d-dimer levels, electrocardiogram (ECG) and chest imaging manifestations. They also reported that the deep learning-based imputation technique performs better than the traditional approach for filling the missing values of clinical data. | Not supplied |
| [24] | India | human liver | Conference | Artificial Neural network (ANN) | 700 | blood samples | They developed a NN based model that diagnoses the liver infection and classifies the type of hepatitis infection as the type of liver infection | Not supplied |
| [25] | China | knee joint | Journal | The signal-to-noise ratio | 2 images | image data | They built a 3D visualization system to model the knee joint motion and supported the analysis of motion trajectories to achieve real-time motion tracking. | Not supplied |
| [26] | Colombia | patients with osteoporosis | Conference | Finite element analysis | 1 | image data | They develop a 3D model from computer axial tomography of a patient to assess the condition of bone tissues affected by osteoporosis. They also used Finite element analysis (FEA) method to provide a better description of the stress and strain fields in a bone structure | Not supplied |
| [27] | UK | patients in general | Journal | graph neural network (GNN) | 1 | blood samples, genes and tissues | They create a DT model based on a graph neural network algorithm that predicts patient conditions (e,g,. blood pressure) using clinical data from multiple levels of anatomy and physiology, such as tissues, cells and organs. | non-government |
| [28] | India | patients with cardiac co-morbidity | Conference |  | 12 | the physiological signals (e.g., Electrocardiography (ECG) and Photoplethysmogram (PPG) | They developed a cardiovascular digital twin model to simulate the consequence of the exercise of a person on various cardiac parameters of medical importance. To that end, they used body-worn sensors to capture real-time ECG signals to forecast exercise levels and compute cardiac variables like left ventricular dynamics, cardiac output, ejection fraction and mean arterial pressure during the exercises. | Not supplied |
| [29] | USA | patients with ischemic stroke | Journal | variational autoencoder model | 1216 | Clinical and demographic characteristics (e.g., creatinine, glucose, red blood cell count, sodium) | They create a DT system that uses a variational autoencoder model to predict the trajectories of related clinical criteria in patients at risk of ischemic stroke | no funding |
| [30] | South Korea | stroke patients | Conference | Support vector machine (SVM) | 123 |  | They developed a DT model built using statistical modelling and predictive ML models. The former is used to identify stroke-impaired EEG biomarkers and their threshold measures, and the latter is used to predict stroke in patients. They showed that certain brain activity measurements such as the Brain Symmetry Index (BSI) are important for classifying stroke patients. | government |
| [31] | France | Human breast | Conference | bio-heat mode | - | sensor-collected data e.g., body temperature, blood pressure | They developed a DT model for breast cancer that uses temperature sensor information collected by portable intelligent devices to detect abnormalities and diagnose breast cancer based on breast skin temperature. | Not supplied |
| [32] | Germany | individuals | Journal | Random forest algorithm | 8 | body temperature, humidity, heart rate variability, limp skin temperature and breathing data | They built a DT model to predict the stress level of individuals using a wearable smart textile with integrated sensors. They used 9 sensors to capture relevant stress metrics and used them for predictive models. They showed that the Random Forest Regressor model obtained the best results from a set of 25 models to predict individual stress levels. | Not supplied |
| [33] | Russia | hypertensive patient | Conference | Probabilistic modelling/stochastic methods | 4521 | clinical data, e.g., blood pressure, age, gender, BMI, smoking status | They used a DT model that simulates a hypertensive patient population to lead the various virtual clinical trials with the aim of probabilistic predicting the blood pressure variability and/or the treatment effectiveness of certain antihypertensive drugs. | Government |
| [34] | Australia | inpatient patients | Conference | Bayesian network | 1014 | clinical data; heart failure, diabetes, stroke, epilepsy, asthma, etc | They proposed a Bayesian network model to predict the fall risk prediction of inpatient patients. They used psychosocial, clinical, demographic, and intrinsic factors associated with the patients in deciding their risk acuity | Not supplied |
| [35] | Canada | human heart | Conference | conventional neural network (CNN) | 200 | data from sensors (body area network), medical records, social networks and external sensors. | They designed a DT model called Cardio Twin for Ischemic Heart Disease (IHD) detection using data collected from sensors (body area network), social networks and medical records. Using the data, they used a CNN algorithm to classify non-myocardial and myocardial conditions. They test the model on a sample of 13420 ECG segments. | Not supplied |
| [36] | UK | severe carotid senosis | Journal | principal component analysis (PCA) | - | Age, height, weight, face video | They built a DT model to predict the severity of carotid stenosis from a video of a human face. To that end, they proposed a head vibration model in the present work that is linked to the forces generated by blood flow with or without occlusion which helps to create a synthetic head vibration data. Also, a computer vision algorithm is adopted to use human face videos. | Government |
| [37] | China | liver tumor | Journal | moving least-square (MLS) algorithm | 2 dogs | CT scan data | They provide a system that relies on augmented reality (AR) methods to provide 3D navigation of heterogeneous target regions to predict the motion of the liver for respiratory compensation. | Government |
| [38] | South Korea | Prostate cancer | Journal | Random forest algorithm | 404 | Clinical data (e.g., clinical T stage, Gleason score, BMI, initial prostate-specific antigen level) | They developed a prediction model-based DT that predicts the prostate cancer (PCa) prognosis by forecasting Pathology and biochemical recurrence (BCR) using ML algorithms. They compare several ML algorithms such as Support Vector Machine (SVM), Logistics Regression (LR), and Random Forest (RF) and show that RF has the best performance. | Government |
| [39] | India | elder patients | Journal | Convolutional Neural Network (CNN) | - |  | They generate a digital twin model for elder individuals to detect irregular events in their daily routine by analysing the physical movements of elderly people. They also used blockchain technology to maintain the record of patients with a higher assurance of security and privacy and also developed a CNN to support the rescheduling decisions. | Not supplied |
| [40] | USA | Crohn’s disease | Journal | mechanistic model | 69 | *patient data; age, weight, BMI *disease data; lesions, biomarkers, duration of Crohn's disease *Treatment history; Drug, responsiveness | They developed a DT model for patients suffering from Crohn's disease which can predict patient progress and the response to given treatment. To that end, they designed a mechanistic model that considers the relationship between inflammation and tissue damage in CD. They reported that the proposed system predicted endoscopic remission and mucosal healing after treatment with vedolizumab for 26 weeks with an overall sensitivity of 80% and 75% and overall specificity of 69% and 70%, respectively. | Industry |
| [41] | China | human heart | Conference | Physiological models | 1 virtual patient | EMG signals. Pacing sequences delivered to patient, catheter locations sensed by the EAM system | They created a platform that identifies heart conditions at the time of the cardiac ablation (CA) procedure. They showed that this system can reduce physician workload during the CA procedure. They used heart models to visualise suspected heart conditions and automatically update new information during the procedure. | Not supplied |
| [42] | China | patients treated by MV | Journal | nonlinear hysteresis loop model (HLM) | 32 | *measured ventilator data; Pressure and flow data *clinical data; sex, age, clinical diagnostic | They proposed a DT model that predicts a patient-specific lung mechanics response to changing Mechanical ventilation (MV) settings, specifically forecasting lung mechanics. They used a nonlinear, physiologically-relevant hysteresis loop model (HLM) that represents lung mechanics | Government |
| [43] | France | human liver | Journal | collocation-based Model Order Reduction (MOR) | 385 external surface meshes of livers | medical images | They proposed a model to personalise the human liver anatomy using biomechanical models. They used MRI and CT scan images to identify and construct the surface and volume of the organs with the help of a collocation-based Model Order Reduction (MOR) approach called Sparse Subspace Learning (SSL). To personalise the method, they used Statistical Shape Analysis to get the shape parametrisation of the liver | Government |
| [44] | China | human brain | Journal | Support vector machine (SVM) and AlexNet algorithms | - | MRI images of brain tumour | They developed a diagnosis and prediction model of brain image fusion using MRI brain tumour images, employing the SVM and AlexNet algorithms. The former algorithm is used for preprocessing of brain image data, while the latter algorithm is used to extract and analyse the brain image features. | Not supplied |
| [45] | France | hindfoot | Journal | 3D models | 5 | CT scan images | They developed a 3D model to investigate the impact of the orientation of the subtalar axis on hindfoot deformities. The model helps to evaluate subtalar joint compensation to angular knee deformity and subtalar axis direction. | Not supplied |
| [46] | Germany | atrial fibrillation | Journal | statistical shape models | 29 | *Clinical data, e.g., BMI, hypertension, Coronary artery disease, Heart failure, LA diameter *MRI images | They developed models to identify the optimal ablation therapy for persistent atrial fibrillation (AF) by integrating clinical data from tomographic imaging and electro-anatomical activation time and voltage maps. | International |
| [47] | Malaysia | respiratory failure patients using mechanical ventilation (MV) | Journal | mathematical modelling | 100 virtual patients | clinical pressure-flow (P- ̇V) data | They developed a model to test and validate different MV treatment selection protocols. They used retrospective clinical pressure-flow (P-V) and integrated Volume (V) data to identify respiratory parameters of patients such as respiratory resistance. After validating the created virtual patients, they defined a virtual trial for the rapid prototyping and development of novel, personalised MV treatment approaches and protocols | no grant |
| [48] | Netherlands | human heart | Journal | mathematical modelling | 9 | echocardiographic data | They create a method to estimate the posterior distribution of regional myocardial tissue properties using echocardiographic deformation imaging. This model is used to predict active and passive tissue properties underlying regional deformation patterns. | government |
| [49] | Italy | human heart | Journal | reduced order modeling | 1 | CT images data | The authors presented a DT model relying on reduced order modelling for a new and fast approach to provide computational analysis of different Modified Blalock–Taussig Shunt (MBTS) complex configurations. This method helps to explore the effect of the shunt’s geometry on the fluid flow and obtain a high-fidelity analysis of a wide range of MBTS shapes. | International |
| [50] | China | heart | Journal | VIKOR algorithm | 100 |  | They proposed a DT structure of a Traditional Chinese medicine (TCM) physical health management that uses a designed training system. They showed that this system can be used to treat and intervene patients with chronic diseases to recover. The system includes an online training system, online education and smoking cessation tips. They compared the results of this method against general Western medicine nursing by experimenting each approach on 50 different patients. The results show that the new method provides better results on patients in terms of blood pressure, TCM syndromes, exercise tolerance, and quality of life | Not supplied |
| [51] | Japan | elder patients | Conference | natural language analysis technology | 2 |  | They developed a DT system that uses elderly person's daily activities and provide specific functions relating to mental health support functions. To that end, they used small sensors installed in the elderly person's house to monitor life behaviours. They connect this system with a setting-type communication robot called "PaPeRo i" which has a highly accurate face and voice recognition ability itself. They also implemented a message destination estimation method using a natural language analysis approach that helps an elder person to send messages to the correct destination without indicating the destination person's information explicitly. Using all of these technologies helps elder persons to interact with the robot and provides an opportunity for the robot to diagnose the patients' dementia, e.g., by asking the elderly persons relevant questions from their daily life that match the persons' daily life. | Government |
| [52] | Germany | airway morthology | Journal | Simulation | 1 | Clinical data, e.g., bronchopulmonary dysplasia (BPD) grade, Gestational age, Days of mechanical ventilation, birth weight | They proposed a DT model that simulates the impacts of different ventilator settings during high-frequency oscillatory ventilation (HFOV) and conventional ventilation (CV) for a preterm infant by creating a model based on personal airway morphology and lung mechanics (LM). To simulate the airflow and gas moved to the distal lung, they created a 3D computational model of infant's lung using MRI images. Using this model they tested four different settings ventilations in the simulated patient lung. | Government |
| [53] | USA | patient CT images | Journal | 3D convolutional neural networks | 10 | CT images | The authors created a DT system called iPhantom that creates patient-specific phantoms using patient medical images through organ segmentation. This system helps assess radiation dose to radiosensitive organs in CT imaging patients. Results confirm that it has the ability to precisely localised most of the organs, including low contrast organs, in CT images, obtaining a high accuracy (<10% organ dose error) across radiosensitive organs. |  |
| [54] | Austria | human electrophysiology (EP) | Journal | mathematical modelling | 12 | MRI data | They developed a DT system of ventricular cardiac electrophysiology (EP) that contains a clinically-compatible novel ECG forward model. This enables the model to manage large amounts of data within a reasonable time scale comparable with clinical workflow. | University, government |
| [55] | Australia | patients metabolism | Journal | optimisation model | 12 | clinical data, e.g., interstitial blood glucose, insulin dose, consumed meal | The authors built a DT framework for patients with type 1 diabetes. The proposed framework uses an optimisation model to find the optimal insulin dosing policies, e.g., dual wave, split bolus and standard bolus. They collect the required data from individuals at home, e.g., interstitial blood glucose sensor data and insulin pump information, and then use a linear model for fitting each patient's glucose dynamics. | government |
| [56] | China | individual patients | conference | Federated Learning algorithm | 920 | clinical data, e.g., chest pain type, blood pressure, serum cholesterol, blood sugar, heart rate | They built a framework by designing a Federated Learning algorithm with two client selection models to improve prediction accuracy. The first client selection algorithm improves efficiency by picking high computing power clients while the second one improves accuracy by selecting reliable clients with high-quality datasets. | government |
| [57] | Germany | human joints | Conference | Memory Polynomial Model | 18 | Inertial Measurement Unit (IMU) sensor data | The authors proposed a memory polynomial model (MPM) that uses the magnitude of the acceleration signal of the Inertial Measurement Unit (IMU) sensors located at the ankle magnitude to estimate the lower limb joint angles. The IMU sensors were placed at the feet, lower legs, and upper legs of the participants. They show that this model can be used for gait kinematic analysis | No mentioned |
| [58] | USA | Skull surgery | Journal |  | 1 | Sensor collected data, e.g., 3D poses of each component | The authors developed Twin-S which is a digital twin framework for skull-based surgeries. It enhances situational awareness by accurately tracking and updating surgical tools and anatomy in real-time with an average error of 1.39mm. It improves surgical views and aims for further accuracy enhancements through vision-based techniques. | university |
| [59] | USA | Wound Management | Journal | Convolutional neural network | - | patients’ demographics, such as gender, medical  history, smoking status, and age. Also, 3D imaging of the wounds | The paper presents a framework for managing chronic wounds using data-driven models to predict wound healing and identify non-healing wounds. The model's outputs demonstrate a similar tissue distribution and area measurements to the actual wounds, indicating its capability to predict the healing progress of chronic wounds, e.g. predicting tissue distribution by accuracy rate of around 74% | No mentioned |
| [60] | Turkey | human mouth | Journal | 3D biomechanical model | 1 | CT data containing cranial (skull) images of a patient, cortical and cancellous bones, disks and fossa | The study develops and validates a digital twin model of the human lower jaw, employing non-linear finite element analysis. It aims to understand mandibular biomechanics and fracture patterns under impact loading to enhance prevention and treatment methods for mandibular fractures and medical devices. | No mentioned |
| [61] | Netherlands | Human heart | Journal | CircAdapt model which is a mathematical model of the human heart and circulation | 45 | Clinical and demographic data, e.g., age, gender, Atrial fibrillation, Ischaemic heart disease, Beta-blocker | They developed virtual biventricular pacing in digital twins of HF patients to predict CRT response. The findings suggest that reducing septal-to-lateral work imbalance can forecast post-CRT LV reverse remodelling. This approach may aid in selecting CRT candidates and optimising CRT delivery. | Government |
| [62] | Singapore | patient’s metabolism | Journal | Regression models | 804 | Clinical and demographic data, e.g., gender, age, BMI, HbA1c, LDL-Cholesterol, Fasting glucose | They developed a Generalized Metabolic Flux Analysis (GMFA) framework to predict the future development of diabetic retinopathy and cataract progression within three years from the baseline time point. Using this framework, they demonstrated its ability to predict the evolution path of metabolic health. Their predictive models achieved an ROC-AUC ranging from 0.79 to 0.95. | Governmet |
| [63] | India | patient’s metabolism | Journal | Machine learning algorithm | 64 | glucose profiles, weight, BMI, duration of diabetes, blood pressure, food intake, sleep pattern | This study examined how digital twin (DT) technology, which personalises nutrition, activity, and sleep for individuals with type 2 diabetes (T2D), impacts glycaemic control and liver health. DT patients showed significantly better HbA1C reduction, higher T2D remission rates, and improved liver function and fat reduction compared to standard care. | Non-government |
| [64] | Germany | Multiple myeloma | Journal | Graph-based network | - | Patient characterisation includes demographic data, medical history, clinical observations, laboratory reports, medical imaging reports, genetic data, as well as treatment history | They proposed a similarity-based multiple myeloma digital twin (MMDT) designed for explainability and interpretability, detailing its four-layer implementation, integration of external evidence, and an initial evaluation scenario using the MMRF CoMMpass database. | University |
| [65] | USA | type 2 diabetes (T2D) | Journal | Machine learning algorithms | ~5,000 | comprehensive self-reported data  coupled with multiomic data (proteomics, metabolomics, clinical  labs) | This study presents a framework for a type 2 diabetes digital twin (T2D DT) to monitor patients, integrate omics-scale data, and predict clinical changes, using machine learning and knowledge graphs primarily, while acknowledging the potential of mechanistic models for individual patient representation, requiring deeper disease and patient understanding. | Government |
| [66] | USA | patients with  aortic stenosis | Journal | deep learning models, e.g., the U-Net architecture | 35 | CT images | They created a pipeline to generate comprehensive digital replicas for aortic stenosis (AS) cases, encompassing the aorta, aortic valve, and calcification, utilising clinical CT images. | Government |
| [67] | Spain | Ventricular Tachycardia | Conference | 3D simulations | 21 | Image data, e.g., LGE-MRI data | This study showed the viability of using fast simplified models to simulate various physiological scenarios for assessing ventricular tachycardia (VT) risk, This can help  in the analysis and stratification of patients with complex  infarct regions, providing a risk of suffering an arrhythmia. | Government |
| [68] | India | Diabetic Retinopathy | Conference | Convolutional neural network | 3662 images | retina images | This paper presents a Digital Twin framework for Diabetic Retinopathy within an IoT and AI-based smart healthcare system. It features a classifier model for clinical severity using EfficientNet, achieving 98.36% accuracy. | Not mentioned |
| [69] | Belgium | Patients with Hyperglycemia | Conference | tri-variate kernel density stochastic model | 616 | Demographics and clinical data, e.g., age, gender, blood glucose, insulin rate | The STAR framework, using a new 3D stochastic model, effectively controls glycemia in critically ill patients by accounting for patient variability. In a study of 616 patients, 77% of blood glucose measurements were in the target range, with no severe hypoglycaemia, ensuring high safety and improved ICU outcomes. | Government |
| [70] | Czech Republic | right ventricular dysfunction | Journal | CircAdapt model which is a mathematical model of the human heart and circulation | 2 | Clinical and imaging data, e.g., age, sex, Cardiac magnetic resonance/cardiac CT scan, Echocardiography | They examined the effects of RV cardiac resynchronisation therapy (RV-CRT) on right ventricular performance. By collecting data from two patients, they customised the CircAdapt model to reflect individual heart and circulation dynamics. The findings indicate that this approach could be useful in selecting suitable candidates for RV-CRT, particularly for those with tetralogy of Fallot (ToF) and other related congenital heart disorders. | Government |
| [71] | India | patient’s metabolism | Journal | The Long–Short Term Memory (LSTM) | 15 | Clinical data, Insulin infusion, meal intake, age sex, weight, BMI, interstitial glucose level | This paper presents a framework designed to tailor treatment based on individual patient data by computing precision insulin infusion to avoid BGL. Through trials involving 15 patients, it enhanced time spent within the optimal range, increasing it from 3-75% to 86-97%, while also decreasing insulin infusion requirements by 14-29%. | Government |
| [72] | Italy | glucose concentration | Journal | Simulation | 100 virtual patients | continuous glucose monitoring (CGM) data, insulin data, and constant carbohydrate data | ReplayBG simulates glucose responses to insulin and carbohydrate treatments. Customised models, built from CGM data, forecast treatment effects. Tested on 100 virtual subjects, it reliably explores T1D treatment impacts, proving robust in real-world case studies. | Not mentioned |
| [73] | USA | cardiac electrophysiology in paediatric patients with congenital  heart disease | Journal | the physics-based mathematical model, along with numerical discretisation | 1 | CT scan images data | They created personalised digital cardiac electrophysiology twins for a paediatric patient with hypoplastic left heart syndrome (HLHS), using detailed models and machine learning for calibration. Validated against clinical data, it enables simulation of various scenarios, aiding in treatment strategy assessment. | Government |
| [74] | Switzerland | carotid bifurcations | Journal | Gmsh; a finite-element mesh generator | 26 | CT scan Imaging data | They used computational fluid dynamics (CFD) and digital twins of carotid bifurcations from CT angiography for non-invasive carotid artery disease (CAD) assessment. With 37 patient-specific models, CFD incorporates Doppler ultrasound (DUS)-derived PSV and a Windkessel model. Agreement with DUS on ICA-PSV is 9% ± 20%, ICC 0.88. Hyperaemic simulations reveal notable pressure drop differences in similar ICA stenoses. | University |
| [75] | UK | patient respiration | Journal | signal processing, and Machine  Learning algorithms | - | patient respiration data collected by sensors | This research introduces ResDT, a groundbreaking DT model that uses Wi-Fi Carrier State Information (CSI), refined signal processing, and ML algorithms to monitor and classify patient respiration into binary and multi-class categories. Multiple preprocessing techniques, including DC-offset removal, smoothing filter, IIR bandpass filters, wavelet decomposition, and PCA, were applied to patient respiration data collected via ESP32 Wi-Fi CSI. In ResDT, elliptic filtering yielded the highest BPM accuracy (87.5%) compared to other methods. | University |
|  |  |  |  |  |  |  |  |  |
| [76] | Palestine | hospital department/unit | Conference | Discrete event simulation | - | sensor collected data, e.g. number of patients in the hospital rooms, patient timestamp arrivals and exit | They proposed a DT framework built on top of DES models to manage hospital services in the hospital units such as radiology/lab departments. DES is fed by the real-time data collected by IoT devices capturing patient flow and resources (e.g., staff). The proposed DT model can be used to help decision-makers to address planning problems such as patient waiting times and the bottlenecks. They build the proposed DT on FlexSim software to test its feasibility using randomly generated data. | Not supplied |
| [77] | France | Emergency department | Conference | Discrete event simulation | - | patient flow-related data, e.g. patient arrivals, total length of stay, number of requested paraclinical examinations per patient | They proposed a DT model for the emergency departments at the time of major crisis such as earthquake or terrorist attacks to optimise the pathway of patient care in those conditions. Using the UML state chart, they provide models for patient flow, and for doctors, nurses, and interns activities. The patient model includes various arrival processes to mimic patient arrivals in both regular and conditional situations. The required data is collected from hospital information system and by interviewing doctors and nurses. The former method used for patient arrivals, total length of stay, and type of patient, while the latter used for each step’s processing times (e.g., triage, imaging). The model validated different scenarios, for example, what actions need to be taken to minimise patient’s length of stay and avoid massive crowding of the system during the exceptional times. | Not supplied |
| [78] | Italy | COVID19 vaccination center | Journal | Discrete event simulation | - | patient flow-related data, e.g. number of patient served per nurse, the time spent by each patient for the sevice, the time spent by patients in the queue, | The authors designed a DT model for a walk-in vaccination centre and its real-time monitoring. They use a DES model to simulate the vaccination process (i.e., patient flow) of real system. The required data is collected through a Near Field Communication (NFC) technology specifically designed for this project. To that end, each operation has a smartphone with an NFC application and each patient have a NFC tag. Each tag is registered through the app and given to the patient at the time of their entrance which captures their entire flow and share the information through the app for analysis. The desired outputs (e.g., the number of patients waiting at each time and the number of patients vaccinated every hour by each nurse) are shared with the decision-makers through a designed dashboard. | Not supplied |
| [79] | Canada | COVID19 test center | Conference | Discrete event simulation | - | sensor-collected data, e.g. patient presence, direction and movement, | They focused on improving the operations of a vaccination test centre in the Montreal region, Canada. They developed a hybrid simulation approach built on AnyLogic software. They used a DES to model the patient flow and the testing process, while they used an ABS to model the patient’s behaviour and the resources. They used RFID readers/sensors to capture patient’s activities, e.g., the specific location of patients in different zones such as waiting area, registration area and testing area. Using the collected data, the simulation model allows the decision makers to adjust their operations to improve the performances including re-assigning the doctors and nurses when it is needed. They also discuss that RFID readers provide multiple benefits as data collectors such as affordable price and their disposability to avoid contamination. | Not supplied |
| [80] | France | hospital department/unit | Journal | Discrete event simulation | - | patient flow-related data, e.g. processing time in different stages, number of patients at each timestamp, resource schedules | The authors explained that designing a DT includes four steps of functionalities: construction, validation, transformation, and deployment. In the construction step, a simulation model of patient flow is constructed after extracting related information such as different types of activities (e.g., registration room and waiting room), the corresponding distance between activities and the processing time in each activity. The constructed simulation model is validated in the next step. These two steps are offline as built using historical data. Next, the validated simulation model transferred from an offline mode to online one using real-time data collected by sensors to monitor and predict the patients’ behaviour. At the final stage, the DT model connected to the hospital, e.g., they installed on the hospitals’ servers. | Not supplied |
| [81] | France | hemodialysis unit | Conference | Discrete event simulation, Agent-based Simulation | - | patient flow-related data, e.g.time spent in dialysis station by assisstance, visiting time ast the dialysis station by each nurse, time spent to (dis)connect the patient and the machine | The authors proposed a digital twin model for the haemodialysis unit of Toronto general hospital, Canada to monitor the spread of Omicron variant of COVID-19 within the unit, since these group of patients need to stay for four hours routinely in the unit for treatment and create a high chance of exposure to the Virus. They proposed a simulation model to simulate haemodialysis unit operations and the movements and contacts between different defined agents (e.g., patients, nurses, clerk, and haemodialysis assistants). To that end, they provided a workflow (i.e., treatment process) for each of the selected agents, considered the schedules of agents’ work and modelled the processing times in different steps, e.g., treatment time and waiting times for patients, and the time needed for nurses to connect the patients to dialysis machines. The proposed simulation approach helps to identify the source of viral transmission and limit its spread inside the unit. The AnyLogic and Unity software are used to build the simulated model and provide a 3D version of unit for medical staff respectively. | Government, University |
| [82] | China | Emergency department | Journal | Discrete event simulation-optimisation | - | sensor-collected data, e.g. patient attributes, arrival mode, the time of triage, registration, consultation, and discharge | The authors focused on building a simulation model, called Multi-fidelity Simulation Modelling (MFSM), that optimises/balances speed and accuracy of simulation models. They combined the idea of simulation algorithms with optimisation methods (e.g., evolutionary algorithms) that automatically find the best simulation modules and parameters that keep the models accurate and fast. They explain that the required data, e.g., patients’ arrival rate and treatment times can be collected using smart devices. A one-year historical data was collected to construct the DES model. The actual and simulated data are compared to validate the model with comparing the actual against the simulated results of length of stay for different group of patients (e.g., critical, urgent, and non-urgent). | Not supplied |
| [83] | USA | intensive care units | Journal | Discrete event simulation, Agent-based Simulation | - | patient data, e.g. patient type, patient arrival rate, patient pathway, clinical resources | They built a simulation model of the ICU. To duplicate the ICU operations/activities, they combine DES and ABS. They proposed a patient flow by observing the system and interviewing the clinicians in the unit. They used multiple input for each model. For example, for DES, they used patient arrival rate, clinical resources/capacity and patient flow using historical data. They also used ABS to capture each patient and their behaviour using information such as patient state transmission (e.g., stable, died) and each task processing time using expert opinions. They also provide a user interface that allows the users to modify the model to a given operating environment, test certain scenarios, and display the simulated outputs such as the hourly patient census, the distribution of discharge. | Government |
| [84] | Austria | patient transportation | Journal | optimisation | - | optimisation related data, e.g. transportation requests, pickup and delivery locations, vehicle locations | They focused on the patient transportation request as a part of an emergency medical service in Vienna, Austria. They modelled the problem as an optimisation problem called dial-a-ride routing problem (DARP) where transport the patients from their home to a medical facility or vice versa. They provide an optimisation algorithm called variable neighbourhood search (VNS) that has ability of continuous re-optimisation (i.e., providing new solution after receiving new requests) for different scenarios. They also featured an Anticipatory method to predict some of the future calls since some of the patient requests happen regularly such as dialysis patients. They tested the provided methods on a historical data of daily operations for 17 months, consisting of 284,905 patient transportation requests. | Government |
| [85] | Italy | Emergency department | Journal | Discrete event simulation | - | patient flow-related data, e.g. triage duration, the chance of patients on stretchers, worsening the condition, procedure duration | They mainly focused on the emergency department at the time of a crisis such as earthquake. Using the historical data, the information gathered from interviewing the healthcare experts, and reviewing the existing emergency, they proposed a DES model for patient flow in post-earthquake conditions. The model includes features such as patient arrival in crisis, available medical equipment, patients’ length of stay, mortality rate, available staffs, and some treatment times (e.g., surgery, MRI, lab procedures). They tested the model using a historical data from a small hospital in Central Italy | University |
| [86] | china | clinical building | Journal | Immersive VR | - |  | Authors focused on onboarding healthcare personals at the time of facility extension or to transition to a new clinical environment. As a case study, they focused on the Cincinnati Children’s Hospital Medical Centre as they recently built a new clinical expansion including a new ICU, and a new ED. To that end, they create an immersive VR of the new buildings with the medical equipment and provide orientation sessions for frontline health care staff (e.g., nurses, respiratory therapy). It benefits them to operate efficiently from day one of transition. | Not supplied |

1. Gillette K, Gsell MA, Bouyssier J, Prassl AJ, Neic A, Vigmond EJ, et al. Automated framework for the inclusion of a his–purkinje system in cardiac digital twins of ventricular electrophysiology. Annals of Biomedical Engineering. 2021;49(12):3143-53.

2. Qi T, Cao Y. Virtual clinical trials: A tool for predicting patients who may benefit from treatment beyond progression with pembrolizumab in non‐small cell lung cancer. CPT: Pharmacometrics & Systems Pharmacology. 2023;12(2):236-49.

3. Chakshu NK, Nithiarasu P. An AI based digital-twin for prioritising pneumonia patient treatment. Proceedings of the Institution of Mechanical Engineers, Part H: Journal of Engineering in Medicine. 2022;236(11):1662-74.

4. Doste R, Lozano M, Jimenez-Perez G, Mont L, Berruezo A, Penela D, et al. Training machine learning models with synthetic data improves the prediction of ventricular origin in outflow tract ventricular arrhythmias. Frontiers in Physiology. 2022:1543.

5. Lai M, Yang H, Gu J, Chen X, Jiang Z, editors. Digital-twin-based Online Parameter Personalization for Implantable Cardiac Defibrillators. 2022 44th Annual International Conference of the IEEE Engineering in Medicine & Biology Society (EMBC); 2022: IEEE.

6. Tardini E, Zhang X, Canahuate G, Wentzel A, Mohamed AS, Van Dijk L, et al. Optimal Treatment Selection in Sequential Systemic and Locoregional Therapy of Oropharyngeal Squamous Carcinomas: Deep Q-Learning With a Patient-Physician Digital Twin Dyad. Journal of medical Internet research. 2022;24(4):e29455.

7. Shamanna P, Joshi S, Shah L, Dharmalingam M, Saboo B, Mohammed J, et al. Type 2 diabetes reversal with digital twin technology-enabled precision nutrition and staging of reversal: a retrospective cohort study. Clinical Diabetes and Endocrinology. 2021;7(1):1-8.

8. Silfvergren O, Simonsson C, Ekstedt M, Lundberg P, Gennemark P, Cedersund G. Digital twin predicting diet response before and after long-term fasting. PLOS Computational Biology. 2022;18(9):e1010469.

9. Shamanna P, Saboo B, Damodharan S, Mohammed J, Mohamed M, Poon T, et al. Reducing HbA1c in type 2 diabetes using digital twin technology-enabled precision nutrition: A retrospective analysis. Diabetes Therapy. 2020;11:2703-14.

10. Hwang S-H, Park J-M, Kim J-H, Shim J-S, Jun W-S. A Case of Full-mouth Rehabilitation using a Digital twin Based on Multi-source Data in a Patient with Vertical Dimension Loss due to Multiple Tooth wear and Fractures. 2022.

11. Lal A, Li G, Cubro E, Chalmers S, Li H, Herasevich V, et al. Development and verification of a digital twin patient model to predict specific treatment response during the first 24 hours of sepsis. Critical care explorations. 2020;2(11).

12. Cho S-W, Byun S-H, Yi S, Jang W-S, Kim J-C, Park I-Y, et al. Sagittal relationship between the maxillary central incisors and the forehead in digital twins of korean adult females. Journal of Personalized Medicine. 2021;11(3):203.

13. Shamanna P, Dharmalingam M, Sahay R, Mohammed J, Mohamed M, Poon T, et al. Retrospective study of glycemic variability, BMI, and blood pressure in diabetes patients in the Digital Twin Precision Treatment Program. Scientific Reports. 2021;11(1):14892.

14. Chakshu NK, Sazonov I, Nithiarasu P. Towards enabling a cardiovascular digital twin for human systemic circulation using inverse analysis. Biomechanics and modeling in mechanobiology. 2021;20(2):449-65.

15. Ahmadian H, Mageswaran P, Walter BA, Blakaj DM, Bourekas EC, Mendel E, et al. Toward an artificial intelligence‐assisted framework for reconstructing the digital twin of vertebra and predicting its fracture response. International Journal for Numerical Methods in Biomedical Engineering. 2022;38(6):e3601.

16. Golse N, Joly F, Combari P, Lewin M, Nicolas Q, Audebert C, et al. Predicting the risk of post-hepatectomy portal hypertension using a digital twin: A clinical proof of concept. Journal of Hepatology. 2021;74(3):661-9.

17. Ahmadian H, Mageswaran P, Walter BA, Blakaj DM, Bourekas EC, Mendel E, et al. A digital twin for simulating the vertebroplasty procedure and its impact on mechanical stability of vertebra in cancer patients. International Journal for Numerical Methods in Biomedical Engineering. 2022;38(6):e3600.

18. Wu C, Jarrett AM, Zhou Z, Elshafeey N, Adrada BE, Candelaria RP, et al. MRI-based digital models forecast patient-specific treatment responses to neoadjuvant chemotherapy in triple-negative breast cancer. Cancer Research. 2022;82(18):3394-404.

19. Talukder AK, Schriml L, Ghosh A, Biswas R, Chakrabarti P, Haas RE. Diseasomics: Actionable machine interpretable disease knowledge at the point-of-care. PLOS Digital Health. 2022;1(10):e0000128.

20. Batch KE, Yue J, Darcovich A, Lupton K, Liu CC, Woodlock DP, et al. Developing a cancer digital twin: Supervised metastases detection from consecutive structured radiology reports. Frontiers in artificial intelligence. 2022;5:26.

21. Aubert K, Germaneau A, Rochette M, Ye W, Severyns M, Billot M, et al. Development of digital twins to optimize trauma surgery and postoperative management. A case study focusing on tibial plateau fracture. Frontiers in Bioengineering and Biotechnology. 2021;9:722275.

22. Hernigou P, Olejnik R, Safar A, Martinov S, Hernigou J, Ferre B. Digital twins, artificial intelligence, and machine learning technology to identify a real personalized motion axis of the tibiotalar joint for robotics in total ankle arthroplasty. International Orthopaedics. 2021;45:2209-17.

23. Tai Y, Zhang L, Li Q, Zhu C, Chang V, Rodrigues JJ, et al. Digital-Twin-Enabled IoMT System for Surgical Simulation Using rAC-GAN. IEEE Internet of Things Journal. 2022;9(21):20918-31.

24. Palaniappan R, Surendran S, editors. A Digital Twin Approach for deepened Classification of Patients with Hepatitis, Fibrosis and Cirrhosis. Journal of Physics: Conference Series; 2022: IOP Publishing.

25. Chen J. 3d visualization analysis of motion trajectory of knee joint in sports training based on digital twin. Computational Intelligence and Neuroscience. 2022;2022.

26. Baena JP, Estrada OG, Villegas D, editors. Structural analysis of bone by segmentation and finite element analysis in patients with osteoporosis. Journal of Physics: Conference Series; 2021: IOP Publishing.

27. Barbiero P, Vinas Torne R, Lió P. Graph representation forecasting of patient's medical conditions: Toward a digital twin. Frontiers in genetics. 2021;12:652907.

28. Roy D, Mazumder O, Khandelwal S, Sinha A, editors. Wearable sensor driven Cardiac model to derive hemodynamic insights during exercise. Proceedings of the Workshop on Body-Centric Computing Systems; 2021.

29. Allen A, Siefkas A, Pellegrini E, Burdick H, Barnes G, Calvert J, et al. A digital twins machine learning model for forecasting disease progression in stroke patients. Applied Sciences. 2021;11(12):5576.

30. Hussain I, Hossain MA, Park S-J, editors. A Healthcare Digital Twin for Diagnosis of Stroke. 2021 IEEE International Conference on Biomedical Engineering, Computer and Information Technology for Health (BECITHCON); 2021: IEEE.

31. Meraghni S, Benaggoune K, Al Masry Z, Terrissa LS, Devalland C, Zerhouni N, editors. Towards digital twins driven breast cancer detection. Intelligent Computing: Proceedings of the 2021 Computing Conference, Volume 3; 2021: Springer.

32. Scheuermann C, Binderberger T, Von Frankenberg N, Werner A, editors. Digital twin: A machine learning approach to predict individual stress levels in extreme environments. Adjunct proceedings of the 2020 ACM international joint conference on pervasive and ubiquitous computing and proceedings of the 2020 ACM international symposium on wearable computers; 2020.

33. Semakova A, Zvartau N. Data-driven identification of hypertensive patient profiles for patient population simulation. Procedia Computer Science. 2018;136:433-42.

34. Ossai C, Wickramasinghe N. A Bayesian Network Model to Establish a Digital Twin Architecture for Superior Falls Risk Prediction. 2021.

35. Martinez-Velazquez R, Gamez R, El Saddik A, editors. Cardio Twin: A Digital Twin of the human heart running on the edge. 2019 IEEE International Symposium on Medical Measurements and Applications (MeMeA); 2019: IEEE.

36. Chakshu NK, Carson J, Sazonov I, Nithiarasu P. A semi‐active human digital twin model for detecting severity of carotid stenoses from head vibration—A coupled computational mechanics and computer vision method. International journal for numerical methods in biomedical engineering. 2019;35(5):e3180.

37. Shi Y, Deng X, Tong Y, Li R, Zhang Y, Ren L, et al. Synergistic Digital Twin and Holographic Augmented-Reality-Guided Percutaneous Puncture of Respiratory Liver Tumor. IEEE Transactions on Human-Machine Systems. 2022;52(6):1364-74.

38. Kim J-K, Lee S-J, Hong S-H, Choi I-Y. Machine-Learning-Based Digital Twin System for Predicting the Progression of Prostate Cancer. Applied Sciences. 2022;12(16):8156.

39. Manocha A, Afaq Y, Bhatia M. Digital Twin-assisted Blockchain-inspired irregular event analysis for eldercare. Knowledge-Based Systems. 2023;260:110138.

40. Venkatapurapu SP, Iwakiri R, Udagawa E, Patidar N, Qi Z, Takayama R, et al. A Computational Platform Integrating a Mechanistic Model of Crohn’s Disease for Predicting Temporal Progression of Mucosal Damage and Healing. Advances in Therapy. 2022;39(7):3225-47.

41. Wu Y, Tang R, Kang E, Jiang Z, editors. Model-based clinical assist system for cardiac ablation. Proceedings of the ACM/IEEE 12th International Conference on Cyber-Physical Systems; 2021.

42. Zhou C, Chase JG, Knopp J, Sun Q, Tawhai M, Möller K, et al. Virtual patients for mechanical ventilation in the intensive care unit. Computer Methods and Programs in Biomedicine. 2021;199:105912.

43. Lauzeral N, Borzacchiello D, Kugler M, George D, Rémond Y, Hostettler A, et al. A model order reduction approach to create patient-specific mechanical models of human liver in computational medicine applications. Computer methods and programs in biomedicine. 2019;170:95-106.

44. Wan Z, Dong Y, Yu Z, Lv H, Lv Z. Semi-supervised support vector machine for digital twins based brain image fusion. Frontiers in Neuroscience. 2021;15:705323.

45. Hernigou P, Safar A, Hernigou J, Ferre B. Subtalar axis determined by combining digital twins and artificial intelligence: influence of the orientation of this axis for hindfoot compensation of varus and valgus knees. International Orthopaedics. 2022;46(5):999-1007.

46. Azzolin L, Eichenlaub M, Nagel C, Nairn D, Sanchez J, Unger L, et al. Personalized ablation vs. conventional ablation strategies to terminate atrial fibrillation and prevent recurrence. Europace. 2023;25(1):211-22.

47. Ang CYS, Lee JWW, Chiew YS, Wang X, Tan CP, Cove ME, et al. Virtual patient framework for the testing of mechanical ventilation airway pressure and flow settings protocol. Computer Methods and Programs in Biomedicine. 2022;226:107146.

48. Van Osta N, Kirkels FP, Van Loon T, Koopsen T, Lyon A, Meiburg R, et al. Uncertainty quantification of regional cardiac tissue properties in arrhythmogenic cardiomyopathy using adaptive multiple importance sampling. Frontiers in physiology. 2021;12:738926.

49. Kardampiki E, Vignali E, Haxhiademi D, Federici D, Ferrante E, Porziani S, et al. The Hemodynamic Effect of Modified Blalock–Taussig Shunt Morphologies: A Computational Analysis Based on Reduced Order Modeling. Electronics. 2022;11(13):1930.

50. Jiang J, Li Q, Yang F. TCM Physical Health Management Training and Nursing Effect Evaluation Based on Digital Twin. Scientific Programming. 2022;2022.

51. Kobayashi T, Fukae K, Imai T, Arai K, editors. Digital Twin Agent for Super-Aged Society. 2022 IEEE International Conference on Consumer Electronics (ICCE); 2022: IEEE.

52. Förster KM, Roth CJ, Hilgendorff A, Ertl‐Wagner B, Flemmer AW, Wall WA. In silico numerical simulation of ventilator settings during high‐frequency ventilation in preterm infants. Pediatric Pulmonology. 2021;56(12):3839-46.

53. Fu W, Sharma S, Abadi E, Iliopoulos A-S, Wang Q, Lo JY, et al. iPhantom: a framework for automated creation of individualized computational phantoms and its application to CT organ dosimetry. IEEE journal of biomedical and health informatics. 2021;25(8):3061-72.

54. Gillette K, Gsell MA, Prassl AJ, Karabelas E, Reiter U, Reiter G, et al. A framework for the generation of digital twins of cardiac electrophysiology from clinical 12-leads ECGs. Medical Image Analysis. 2021;71:102080.

55. Goodwin GC, Seron MM, Medioli AM, Smith T, King BR, Smart CE. A systematic stochastic design strategy achieving an optimal tradeoff between peak BGL and probability of hypoglycaemic events for individuals having type 1 diabetes mellitus. Biomedical Signal Processing and Control. 2020;57:101813.

56. Yuan X, Zhang J, Luo J, Chen J, Shi Z, Qin M, editors. An Efficient Digital Twin Assisted Clustered Federated Learning Algorithm for Disease Prediction. 2022 IEEE 95th Vehicular Technology Conference:(VTC2022-Spring); 2022: IEEE.

57. Alcaraz JC, Moghaddamnia S, Fuhrwerk M, Peissig J, editors. Efficiency of the memory polynomial model in realizing digital twins for gait assessment. 2019 27th European Signal Processing Conference (EUSIPCO); 2019: IEEE.

58. Shu H, Liang R, Li Z, Goodridge A, Zhang X, Ding H, et al. Twin-S: a digital twin for skull base surgery. International Journal of Computer Assisted Radiology and Surgery. 2023;18(6):1077-84.

59. Sarp S, Kuzlu M, Zhao Y, Gueler O. Digital twin in healthcare: a study for chronic wound management. IEEE Journal of Biomedical and Health Informatics. 2023.

60. Demir O, Uslan I, Buyuk M, Salamci MU. Development and validation of a digital twin of the human lower jaw under impact loading by using non-linear finite element analyses. Journal of the Mechanical Behavior of Biomedical Materials. 2023;148:106207.

61. Koopsen T, Gerrits W, van Osta N, van Loon T, Wouters P, Prinzen FW, et al. Virtual pacing of a patient’s digital twin to predict left ventricular reverse remodelling after cardiac resynchronization therapy. Europace. 2024;26(1):euae009.

62. Batagov A, Dalan R, Wu A, Lai W, Tan CS, Eisenhaber F. Generalized metabolic flux analysis framework provides mechanism-based predictions of ophthalmic complications in type 2 diabetes patients. Health Information Science and Systems. 2023;11(1):18.

63. Joshi S, Shamanna P, Dharmalingam M, Vadavi A, Keshavamurthy A, Shah L, et al. Digital Twin Enabled Personalized Nutrition Improves Metabolic Dysfunction-Associated Fatty Liver Disease in Type 2 Diabetes: Results of a 1-Year Randomized Controlled Study. Endocrine Practice. 2023.

64. Grieb N, Schmierer L, Kim HU, Strobel S, Schulz C, Meschke T, et al. A digital twin model for evidence-based clinical decision support in multiple myeloma treatment. Frontiers in Digital Health. 2023;5:1324453.

65. Zhang Y, Qin G, Aguilar B, Rappaport N, Yurkovich JT, Pflieger L, et al. A framework towards digital twins for type 2 diabetes. Frontiers in Digital Health. 2024;6:1336050.

66. Rouhollahi A, Willi JN, Haltmeier S, Mehrtash A, Straughan R, Javadikasgari H, et al. Cardiovision: a fully automated deep learning package for medical image segmentation and reconstruction generating digital twins for patients with aortic stenosis. Computerized Medical Imaging and Graphics. 2023;109:102289.

67. Serra D, Franco P, Romero P, Romitti G, García-Fernández I, Lozano M, et al., editors. Assessment of Risk for Ventricular Tachycardia based on Extensive Electrophysiology Simulations. 2023 45th Annual International Conference of the IEEE Engineering in Medicine & Biology Society (EMBC); 2023: IEEE.

68. Chahal Y, Tokas R, Sharma K, editors. Smart Solution Using Digital Twin and IoT for Diabetic Retinopathy. 2023 14th International Conference on Computing Communication and Networking Technologies (ICCCNT); 2023: IEEE.

69. Uyttendaele V, Knopp JL, Desaive T, Chase JG. Clinical trial validation of the STAR-3D glycemic control framework. IFAC-PapersOnLine. 2023;56(2):4758-63.

70. Ložek M, Kovanda J, Kubuš P, Vrbík M, Lhotská L, Lumens J, et al. How to assess and treat right ventricular electromechanical dyssynchrony in post-repair tetralogy of Fallot: insights from imaging, invasive studies, and computational modelling. Europace. 2024;26(2):euae024.

71. Thamotharan P, Srinivasan S, Kesavadev J, Krishnan G, Mohan V, Seshadhri S, et al. Human digital twin for personalized elderly type 2 diabetes management. Journal of Clinical Medicine. 2023;12(6):2094.

72. Cappon G, Vettoretti M, Sparacino G, Del Favero S, Facchinetti A. Replaybg: A digital twin-based methodology to identify a personalized model from type 1 diabetes data and simulate glucose concentrations to assess alternative therapies. IEEE Transactions on Biomedical Engineering. 2023.

73. Salvador M, Kong F, Peirlinck M, Parker DW, Chubb H, Dubin AM, et al. Digital twinning of cardiac electrophysiology for congenital heart disease. Journal of the Royal Society Interface. 2024;21(215):20230729.

74. Dubs L, Charitatos V, Buoso S, Wegener S, Winklhofer S, Alkadhi H, et al. Assessment of extracranial carotid artery disease using digital twins–A pilot study. NeuroImage: Clinical. 2023;38:103435.

75. Khan S, Alzaabi A, Iqbal Z, Ratnarajah T, Arslan T. A Novel Digital Twin (DT) model based on WiFi CSI, Signal Processing and Machine Learning for patient respiration monitoring and decision-support. IEEE Access. 2023.

76. Karakra A, Fontanili F, Lamine E, Lamothe J, Taweel A, editors. Pervasive computing integrated discrete event simulation for a hospital digital twin. 2018 IEEE/ACS 15th international conference on computer systems and Applications (AICCSA); 2018: IEEE.

77. Augusto V, Murgier M, Viallon A, editors. A modelling and simulation framework for intelligent control of emergency units in the case of major crisis. 2018 winter simulation conference (WSC); 2018: IEEE.

78. Pilati F, Tronconi R, Nollo G, Heragu SS, Zerzer F. Digital twin of COVID-19 mass vaccination centers. Sustainability. 2021;13(13):7396.

79. Maïzi Y, Bendavid Y, editors. Designing a RFID/IoT prototype for improving COVID19 test centers daily operations. 20th International Conference on Modeling and Applied Simulation, MAS 2021; 2021.

80. Karakra A, Fontanili F, Lamine E, Lamothe J. A discrete event simulation-based methodology for building a digital twin of patient pathways in the hospital for near real-time monitoring and predictive simulation. Digital Twin. 2022;2(1):1.

81. Possik J, Azar D, Solis AO, Asgary A, Zacharewicz G, Karami A, et al., editors. A distributed digital twin implementation of a hemodialysis unit aimed at helping prevent the spread of the Omicron COVID-19 variant. 2022 IEEE/ACM 26th International Symposium on Distributed Simulation and Real Time Applications (DS-RT); 2022: IEEE.

82. Chen W, Hong W, Zhang H, Yang P, Tang K. Multi-Fidelity Simulation Modeling for Discrete Event Simulation: An Optimization Perspective. IEEE Transactions on Automation Science and Engineering. 2022.

83. Zhong X, Babaie Sarijaloo F, Prakash A, Park J, Huang C, Barwise A, et al. A multidisciplinary approach to the development of digital twin models of critical care delivery in intensive care units. International Journal of Production Research. 2022;60(13):4197-213.

84. Ritzinger U, Puchinger J, Rudloff C, Hartl RF. Comparison of anticipatory algorithms for a dial-a-ride problem. European Journal of Operational Research. 2022;301(2):591-608.

85. Basaglia A, Spacone E, van de Lindt JW, Kirsch TD. A discrete-event simulation model of hospital patient flow following major earthquakes. International Journal of Disaster Risk Reduction. 2022;71:102825.

86. Zackoff MW, Rios M, Davis D, Boyd S, Roque I, Anderson I, et al. Immersive Virtual Reality Onboarding using a Digital Twin for a New Clinical Space Expansion: A Novel Approach to Large-Scale Training for Health Care Providers. The Journal of Pediatrics. 2023;252:7-10. e3.
