# Supplementary material for: Digital Twins for Clinical and Operational Decision-Making: Scoping Review
Source: J Med Internet Res. 2025 Jan 8;27:e55015. doi: 10.2196/55015 (PMC11754991; doi:10.2196/55015)
Supplement: Multimedia Appendix 3 [file jmir_v27i1e55015_app3.docx]

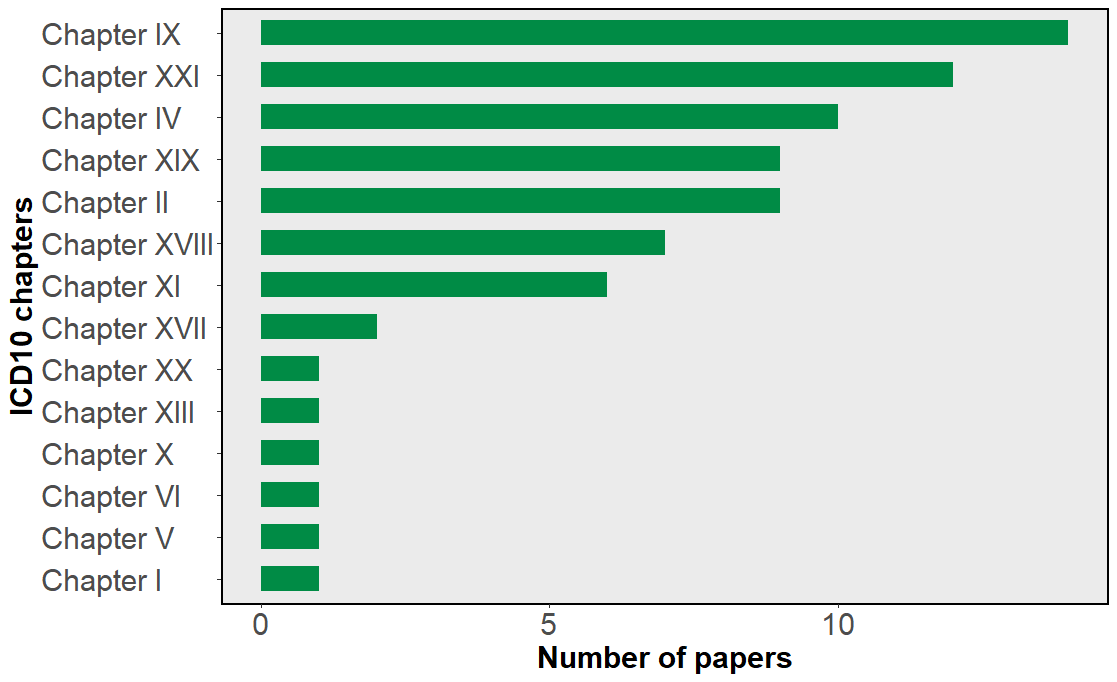


Distribution of disease areas in the included papers using ICD-10 codes


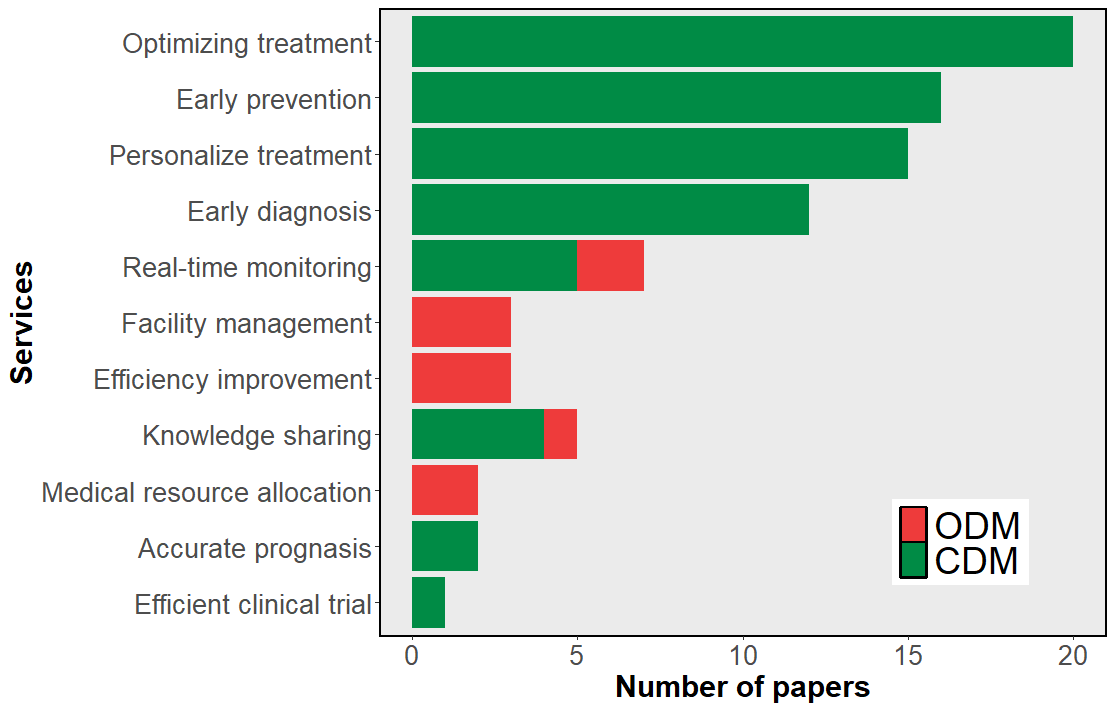


Distribution of healthcare services in the included studies


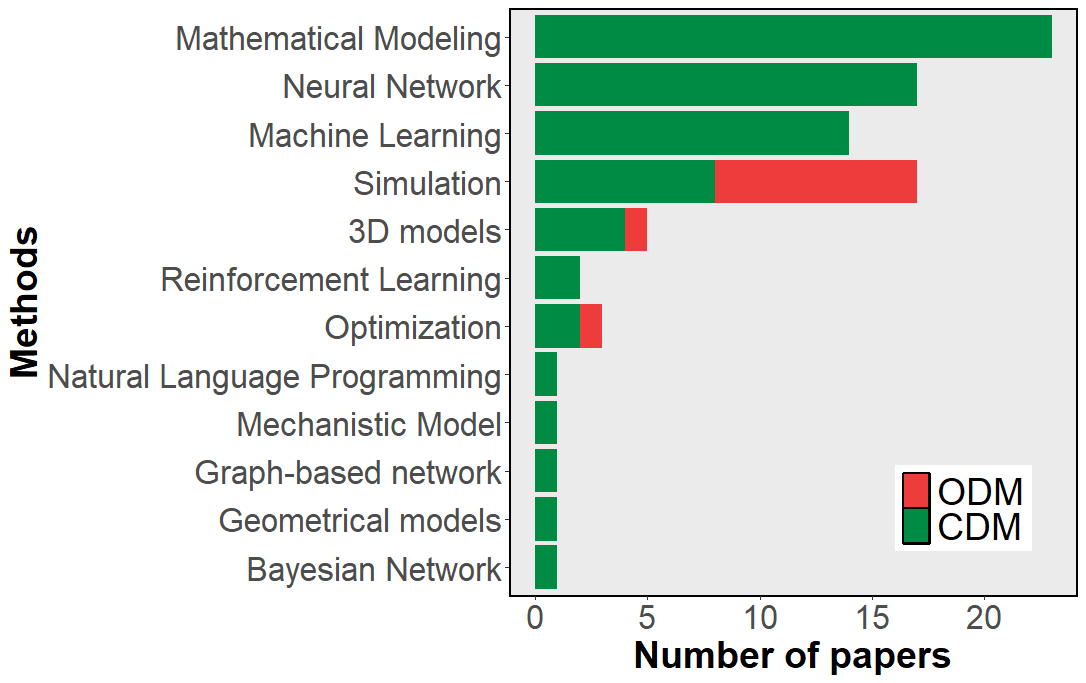


Distribution of computational methods used in the included papers
